# Supplementary material for: Treatments for gestational diabetes: a systematic review and meta-analysis
Source: BMJ Open. 2017 Jun 24;7(6):e015557. doi: 10.1136/bmjopen-2016-015557 (PMC5734427; doi:10.1136/bmjopen-2016-015557)
Supplement: Supplementary material 1 [file bmjopen-2016-015557supp001.pdf]

Supplementary file 1: Search strategy

1 exp diabetes, gestational/ (8715)
2 (gestation$ adj4 diabet$).ti,ab. (10162)
3 gdm.ti,ab. (4203)
4 (glucose adj4 (pregnan$ or gestation$ or prenatal$ or antenatal$ or pre-natal$ or ante-natal$ or maternal$)).ti,ab. (3796)
5 or/1-4 (15126)
6 Glucose Intolerance/ (7142)
7 Glucose Tolerance Test/ (31300)
8 IGT.ti,ab. (4074)
9 ((impair$ or reduced) adj2 glucose).ti,ab. (19442)
10 (glucose adj (tolerance$ or intolerance$)).ti,ab. (40791)
11 (gtt or ogtt).ti,ab. (7907)
12 Prediabetic State/ (4763)
13 (prediabet$ or pre-diabet$).ti,ab. (6103)
14 exp Insulin Resistance/ (64450)
15 (metabolic syndrome$ or syndrome$ x or borderline diabet$).ti,ab. (37636)
16 or/6-15 (134039)
17 exp Pregnancy/ (795751)
18 (pregnan$ or gestation$ or prenatal$ or antenatal$ or pre-natal$ or ante-natal$ or maternal$).ti,ab. (639369)
19 or/17-18 (1008161)
20 16 and 19 (10229)
21 5 or 20 (20405)
22 randomized controlled trial.pt. (421926)
23 controlled clinical trial.pt. (91079)
24 random$.ti,ab. (841233)
25 placebo.ti,ab. (176519)
26 drug therapy.fs. (1876752)
27 trial.ti,ab. (430134)
28 groups.ab. (1574965)
29 or/22-28 (3970247)
30 21 and 29 (6337)
31 (2014$ or 2015$ or 2016$).ed,dc,dp,ep,vd,yr. (3346601)
32 30 and 31 (1671)
33 animals/ not humans/ (4235813)
34 32 not 33 (1555)
