# Supplementary material for: Treatments for gestational diabetes: a systematic review and meta-analysis
Source: BMJ Open. 2017 Jun 24;7(6):e015557. doi: 10.1136/bmjopen-2016-015557 (PMC5734427; doi:10.1136/bmjopen-2016-015557)
Supplement: Supplementary Table 1 [file bmjopen-2016-015557supp002.pdf]

Supplementary Table 1: Quality assessment of the included trials

Author	Year	Random sequence generation	Allocation concealment	Blinding of participants	Blinding of outcome assessments	Completeness of outcome data	Selective reporting
Ainuddin[1]	2015	unclear	high risk	high risk	unclear	low risk	unclear
Anjalakshi[2]	2007	unclear	unclear	unclear	unclear	low risk	unclear
Asemi[3]	2014	low risk	unclear	high risk	high risk	low risk	low risk
Bertini[5]	2005	low risk	low risk	high risk	high risk	low risk	low risk
Bevier[6]	1999	unclear	unclear	high risk	high risk	high risk	low risk
Bonomo[7]	2005	unclear	unclear	high risk	high risk	low risk	unclear
Crowther[8]	2005	low risk	low risk	high risk	low risk	low risk	low risk
Cypryk[9]	2007	unclear	high risk	unclear	unclear	low risk	high risk
Deveer[10]	2013	high risk	high risk	high risk	high risk	low risk	low risk
Elnour[11]	2008	unclear	high risk	high risk	high risk	high risk	low risk
Fadl[12]	2015	Low risk	low risk	unclear	unclear	low risk	unclear
Garner[13]	1997	low risk	high risk	high risk	high risk	low risk	low risk
George[14]	2015	low risk	high risk	high risk	unclear	low risk	low risk

Author	Year	Random sequence generation	Allocation concealment	Blinding of participants	Blinding of outcome assessments	Completeness of outcome data	Selective reporting
Hague[15]	2003	unclear	unclear	unclear	unclear	unclear	unclear
Hassan[16]	2012	high risk	high risk	unclear	unclear	low risk	low risk
Ijas[17]	2010	low risk	low risk	high risk	high risk	low risk	low risk
Lain[18]	2009	low risk	low risk	low risk	low risk	high risk	low risk
Landon[19]	2009	low risk	low risk	high risk	low risk	low risk	low risk
Langer[20]	2000	low risk	unclear	unclear	unclear	low risk	low risk
Li[21]	1987	high risk	unclear	high risk	unclear	low risk	low risk
Louie[22]	2011	low risk	low risk	low risk	unclear	low risk	high risk
Ma[23]	2015	high risk	high risk	high risk	unclear	low risk	unclear
Mesdaghinia[24]	2012	low risk	low risk	low risk	low risk	low risk	low risk
Mirzamoradi[25]	2015	unclear	unclear	high risk	unclear	low risk	unclear
Moore[26]	2007	low risk	unclear	unclear	unclear	low risk	low risk
Moore[27]	2010	low risk	low risk	high risk	high risk	low risk	low risk
Moreno-Castilla[28]	2013	unclear	low risk	high risk	unclear	low risk	low risk
Mukhopadhyay[29]	2012	low risk	unclear	unclear	unclear	low risk	low risk

Author	Year	Random sequence generation	Allocation concealment	Blinding of participants	Blinding of outcome assessments	Completeness of outcome data	Selective reporting
Niromanesh[30]	2012	low risk	low risk	unclear	low risk	low risk	low risk
Ogunyemi[31]	2007	low risk	unclear	unclear	unclear	low risk	unclear
O'Sullivan[32]	1966	unclear	unclear	high risk	high risk	unclear	unclear
Rae[33]	2000	unclear	unclear	low risk	unclear	low risk	high risk
Rowan[34]	2008	low risk	unclear	high risk	high risk	low risk	low risk
Silva[35]	2012	low risk	unclear	high risk	high risk	low risk	low risk
Silva[36]	2007	unclear	low risk	high risk	high risk	low risk	low risk
Spaulonci[37]	2013	low risk	unclear	unclear	unclear	low risk	low risk
Tempe[38]	2013	unclear	unclear	unclear	unclear	low risk	low risk
Tertti[39]	2013	unclear	unclear	unclear	unclear	low risk	low risk
Yang[40]	2014	unclear	high risk	low risk	high risk	low risk	unclear
Yang[41]	2003	unclear	unclear	high risk	unclear	high risk	unclear
Yao[42]	2015	unclear	unclear	unclear	unclear	Low risk	unclear
Zinnat[43]	2013	unclear	unclear	unclear	unclear	low risk	unclear

▲= Alwan review- publications identified by their 2011 search and awaiting classification
