# Supplementary figures and images for: Treatments for gestational diabetes: a systematic review and meta-analysis
Source: BMJ Open. 2017 Jun 24;7(6):e015557. doi: 10.1136/bmjopen-2016-015557 (PMC5734427; doi:10.1136/bmjopen-2016-015557)

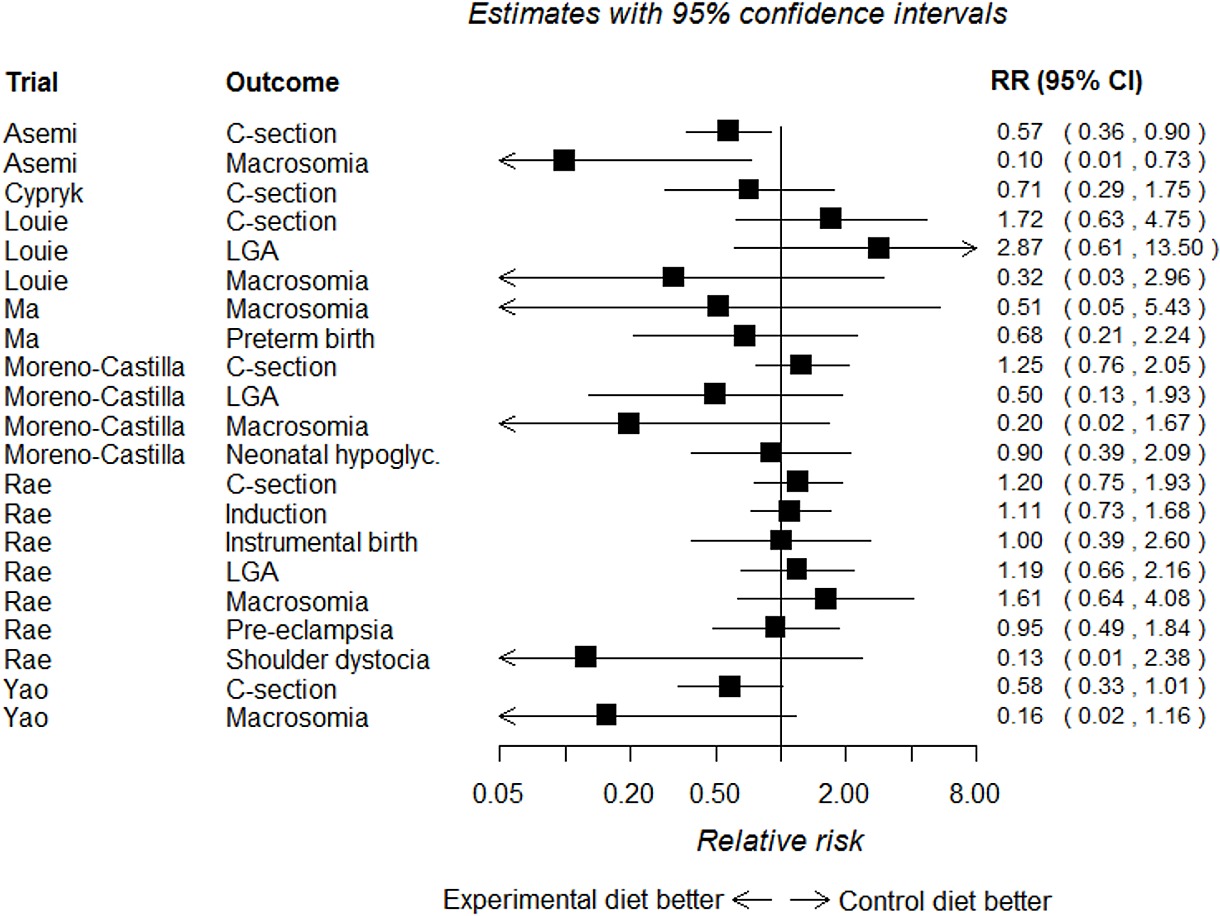

Supplement: Supplementary Figure 1 [file bmjopen-2016-015557supp003.jpg]

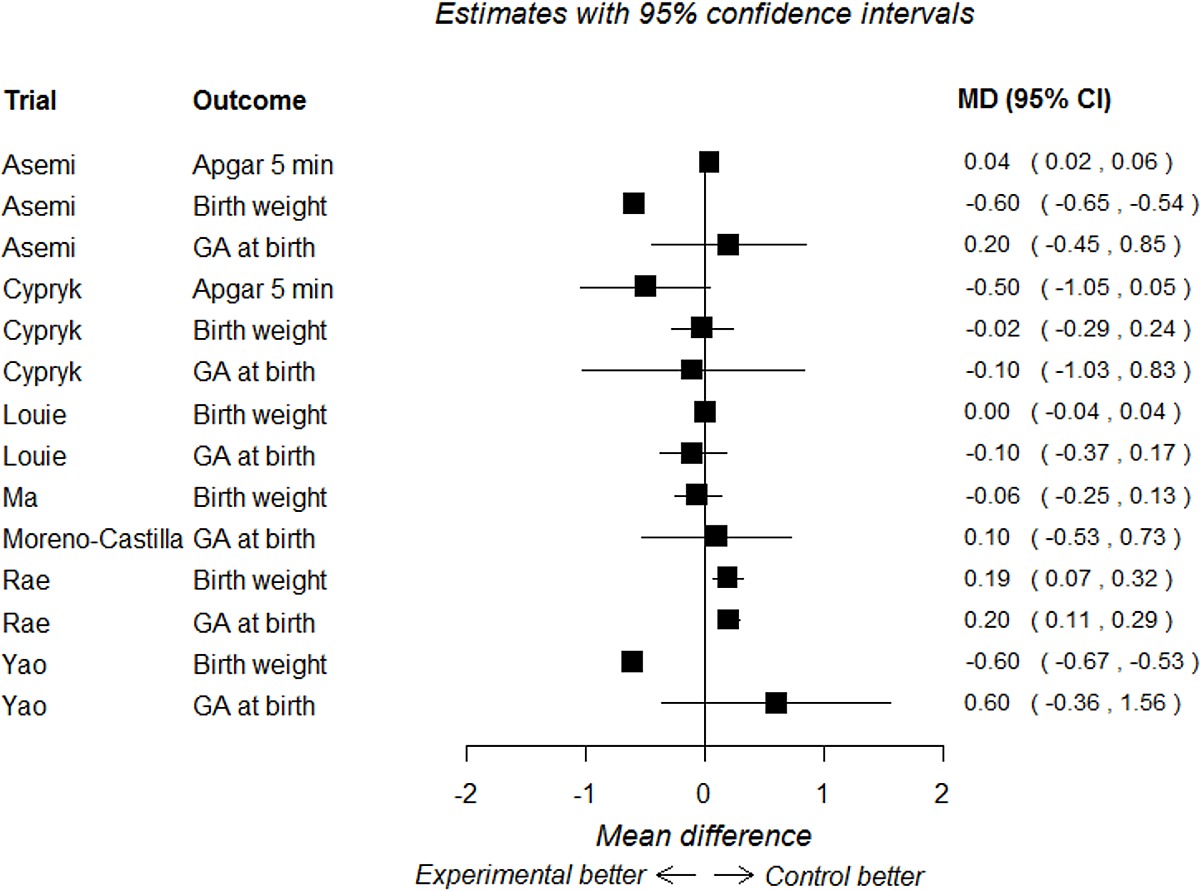

Supplement: Supplementary Figure 2 [file bmjopen-2016-015557supp004.jpg]
