# Supplementary material for: Treatments for gestational diabetes: a systematic review and meta-analysis
Source: BMJ Open. 2017 Jun 24;7(6):e015557. doi: 10.1136/bmjopen-2016-015557 (PMC5734427; doi:10.1136/bmjopen-2016-015557)
Supplement: Author's manuscript [file bmjopen-2016-015557.draft_revisions.pdf]

BMJ Open

Treatments for gestational diabetes: A systematic review and meta-analysis

Journal:	*BMJ Open*
Manuscript ID	bmjopen-2016-015557
Article Type:	Research
Date Submitted by the Author:	13-Dec-2016
Complete List of Authors:	farrar, diane; Bradford Institute for Health research, Maternal and Child health<br>Simmonds, Mark; University of York, Centre for Reviews and Dissemination<br>Bryant , Maria ; University of Leeds,<br>Sheldon, Trevor; University of York, health Sciences<br>tuffnell, derek; Bradford Royal Infirmary, Women's and Newborn unit<br>Golder, Su; University of York, Department of Health Sciences<br>Lawlor, Debbie; Department of Social Medicine, University of Bristol, MRC Integrative Epidemiology Unit
**Primary Subject Heading**:	Obstetrics and gynaecology
Secondary Subject Heading:	Diabetes and endocrinology
Keywords:	gestational diabetes, packages of care, insulin, metformin, glibenclamide, systematic review

SCHOLARONE™
Manuscripts

Treatments for gestational diabetes: A systematic review and meta-analysis

Diane Farrar*, Bradford Institute for Health Research, Bradford Royal Infirmary and Department of Health Sciences, University of York

Mark Simmonds, Centre for Reviews and Dissemination, University of York

Maria Bryant, Leeds Institute of Clinical Trials Research, University of Leeds

Trevor A Sheldon, Hull York Medical School, University of York

Derek Tuffnell, Bradford Women's and Newborn Unit, Bradford

Su Golder, Department of Health Sciences, University of York

Debbie A Lawlor, MRC Integrative Epidemiology Unit and School of Social and Community Medicine at the University of Bristol

*Corresponding author

Email: Diane.Farrar@bthft.nhs.uk

Tel: 01274 383416

Abstract word count: 371

Main text word count: 3723

Running title: Treatments for gestational diabetes

For peer review only - http://bmjopen.bmj.com/site/about/guidelines.xhtml

Abstract

Objective: To investigate the effectiveness of different treatments for GDM.

Design: Systematic review, meta-analysis and network-analysis

Methods: Data sources were searched up to July 2016 and included: MEDLINE and Embase. Randomised trials comparing treatments for GDM (packages of care (dietary and lifestyle interventions with pharmacological treatments as required), insulin, metformin, glibenclamide (glyburide)), were selected by two authors and double checked for accuracy. Outcomes included: large for gestational age; shoulder dystocia; neonatal hypoglycaemia; Caesarean section and pre-eclampsia. We pooled data using random-effects meta-analyses and used Bayesian network meta-analysis to compare pharmacological treatments (i.e. including treatments not directly compared within a trial).

Results: Forty two trials were included, the reporting of which was generally poor with unclear or high risk of bias. Packages of care varied in their composition and reduced the risk of most adverse perinatal outcomes compared to routine care (e.g. large for gestational age: RR 0.58 (95% CI 0.49-0.68; I^2 =0%; trials=8; participants =3462). Network meta-analyses suggest metformin had the highest probability of being the most effective in reducing the risk of most outcomes compared to insulin or glibenclamide.

Conclusions: Evidence shows packages of care are effective in reducing the risk of most adverse perinatal outcomes. However trials are often poorly reported with unclear or high risk of bias. Large well-designed and conducted trials are urgently needed.

Systematic Review Registration: PROSPERO CRD42013004608

Key words: gestational diabetes; systematic review; meta-analysis; network analysis, treatments; packages of care; insulin; metformin; glibenclamide (glyburide)

For peer review only - http://bmjopen.bmj.com/site/about/guidelines.xhtml

Strengths and limitations of this study:

This systematic review evaluates available interventions for the treatment of gestational hyperglycaemia and includes a network-analysis comparing all pharmacological treatments for gestational diabetes.

A large number of trials conducted in varied populations have been included.

For some comparisons the numbers of trials included were few and outcomes reported were few.

Trial quality was generally poor with subsequent high or unclear risk of bias.

For peer review only

For peer review only - http://bmjopen.bmj.com/site/about/guidelines.xhtml

Introduction

Treatment of gestational diabetes (GDM) aims to reduce hyperglycaemia and in turn reduce the risk of adverse perinatal outcomes including: large for gestational age (LGA), macrosomia, shoulder dystocia, neonatal hypoglycaemia and the need for Caesarean section. Diet modification is often used as first-line treatment and if partly or wholly unsuccessful, or where women have substantially elevated glucose at diagnosis, pharmacological treatments (metformin, glibenclamide (glyburide) and/or insulin) are offered.

Previous systematic reviews have investigated the effectiveness of treatments for GDM,[1-11] Although results from these reviews generally indicate that treatments reduce the risk of adverse perinatal outcomes, the searches have variable inclusion criteria and were undertaken between 2009[1,5] and 2014[2-4,7,12 11 6,8,10,12] with just one review with searches in 2015[9] and since then several trials have been published and recommended criteria for GDM diagnosis has changed. Some reviews have included observational studies and most do not review all treatments, with the exception of the Cochrane treatments review[1] (which is now out of date and has been divided for future updates) and the UK NICE guideline.[12] Consequently most previous reviews do not provide an assessment of all available treatments and most have not used a network meta-analysis to determine the most effective pharmacological treatment across all alternatives included in any randomised trial (RCT).

The aim of this study was to systematically review, and where appropriate pool all results from RCTs of the effect of any treatment on GDM and to determine which treatment is most effective.

Methods

We conducted a systematic review, meta- and network-analysis to evaluate whether treatments for GDM reduce the risks of adverse perinatal outcomes and to compare the effectiveness of these treatments.

For peer review only - http://bmjopen.bmj.com/site/about/guidelines.xhtml

This review and meta-analysis was conducted in accordance with Cochrane systematic reviews[13] and the Centre for Reviews and Dissemination recommendations,[14] we have reported our findings following the PRISMA reporting guidelines.[15] This review forms part of a larger Health Technology Assessment report of the diagnosis and management of GDM.[16]

Patient involvement

The outcomes we included were from the Cochrane Pregnancy and Childbirth Group's standardised outcomes for reviews of diabetes in pregnancy. Women who had experienced or had the potential to experience GDM contribute to the design and appraisal of this group's methods and reviews and therefore have influenced the design of this review and outcomes examined.[17]

Search methods

The search strategies were designed to identify records of RCTs of treatment for women with GDM, added to search sources since the search date (July 2011, trials awaiting classification) of the Cochrane 'treatments for GDM' review.[1] The bibliographic databases searched were MEDLINE and MEDLINE in Process, Embase and the Cochrane Central Register of Controlled Trials. Strategies were not restricted by language and were developed using a combination of subject indexing terms and free text search terms in the title and abstract fields. Searches were first conducted in September 2013 and updated in October 2014 and 6th July 2016 using the same search strategies. Information on studies in progress was sought by searching relevant trial registers including ClinicalTrials.gov.

We also searched previously published systematic reviews to ensure any eligible RCTs from these were included in our review if eligible.[2-9] In addition we checked the references of included journal articles. An example of search terms for MEDLINE are included in Supplementary file 1.

For peer review only - http://bmjopen.bmj.com/site/about/guidelines.xhtml

Study selection: Inclusion and exclusion criteria

We included RCTs in which women with diagnosed GDM or impaired glucose tolerance (IGT) (using any definition) were randomised to a treatment designed to lower blood glucose (pharmacological or dietary modification) compared to routine antenatal care (however defined by the trial) or another treatment. Trials including women with pre-existing diabetes were excluded. Trials had to report effects on adverse perinatal outcomes. Included outcomes (defined in any way by the trials) were: gestational age at birth; birth weight (BW); macrosomia; large for gestational age (LGA); shoulder dystocia; preterm birth (less than 37 weeks gestation); neonatal hypoglycaemia; admission to neonatal intensive care unit (NICU); Caesarean section (elective or emergency); pre-eclampsia; pregnancy-induced hypertension (PIH); induction of labour; instrumental birth (forceps or ventouse); Apgar score at five minutes; and negative treatment effects (e.g. gastrointestinal upset, wellbeing). Data on side effects and quality of life measures were also examined. Conference abstracts and letters to journals were eligible for inclusion if they reported sufficient outcome data.

Data extraction and risk of bias assessment

Title and abstract screening and then full text screening was performed by two reviewers (DF, MS, MB or SG) with disagreements resolved by consensus or by the third reviewer. The risk of bias of the included trials was assessed using the Cochrane risk of bias tool,[18] which considers: sequence generation, allocation concealment, blinding of participants and medical staff to treatment allocation, blinding of assessors, loss to follow up, selective reporting of outcomes and other sources of bias. Each criterion was classified as being at low or high risk of bias, or unclear. Two reviewers independently assessed all criteria (DF, MS or SG).

For peer review only - http://bmjopen.bmj.com/site/about/guidelines.xhtml

Statistical analysis

Trials were divided into categories according to the included treatments: (1) insulin versus metformin; (2) insulin versus glibenclamide (glyburide); (3) metformin versus glibenclamide; (4) packages of care: diet or dietary advice with or without exercise or glucose monitoring, with or without supplemental metformin, glibenclamide or insulin, compared to routine antenatal care; (5) comparisons of different dietary modifications.

For dichotomous outcomes, the relative risk comparing each group, with its 95% confidence interval, was calculated from the numbers of outcome events in each randomised group and the number randomised to each group. For continuous outcomes, the difference in means between groups was calculated from the mean and standard deviation of the outcome. For each outcome, and within each of the treatment categories, relative risks or differences in means were pooled in random-effects DerSimonian-Laird meta-analyses.[19] Heterogeneity was assessed using I^2.[20] Analyses were performed to investigate differences in risk of outcomes across varying degrees of hyperglycaemia (defined by a positive/negative GDM screening and diagnostic test). Because of the large number of treatments and outcome comparisons, pooled estimates only are presented in the main paper. Tests for publication bias were considered, but not performed, because there were insufficient trials in any meta-analysis for such tests to be reliable.

We conducted a network meta-analysis to combine information across multiple treatments simultaneously, this combines direct and indirect data to improve the estimation of the effectiveness of treatments and specifically to try to estimate which is the most effective of a number of different treatment options.[21] Analyses were undertaken for each dichotomous outcome using a Bayesian approach, based on the models originally created by Lu and Ades,[22] using the OpenBUGS[23] software. Each model generated a comparison between treatments, expressed as an odds ratio, and percentage indicating the probability that the treatment was the best treatment to

For peer review only - http://bmjopen.bmj.com/site/about/guidelines.xhtml

reduce the incidence of the adverse outcome. This approach was not possible for continuously measured outcomes and so was not undertaken for gestational age, birthweight and Apgar score. As there were no trials comparing diet modification to pharmacological treatments, diet modification could not be included in the network meta-analyses.

Results

Details of included and excluded trials

12234 citations were identified by the original and the two update searches. These citations were combined with three additional citations identified by previous systematic reviews conducted prior to our first searches .[1-5] Following de-duplication and inclusion of additional records, 6437 citations were reviewed. Of these, 214 were judged potentially eligible based on title and abstract. After obtaining the full text publications and assessing eligibility, 42 trials were included and 35 of these were combined in at least one meta-analysis (Figure 1).

Having extracted data from the RCTs assessing packages of care and dietary intervention comparisons (Table 1), we decided that it was not appropriate to pool results from trials comparing dissimilar dietary modification interventions (Table 1). Packages of care included various combinations of interventions, however all packages of care compared with routine care trial results were pooled in meta-analyses.

We included nine publications not included in any previous published review. Two compared metformin and insulin,[24,25] one, glibenclamide and insulin,[26] four, packages of care with routine care[27-30] and two compared different dietary modification interventions.[31,32] Seven of these trials were reported after the search dates of the previous reviews and were published in 2014 or 2015, the remaining two trials (dietary modification interventions or packages of care) did not fulfil other

For peer review only - http://bmjopen.bmj.com/site/about/guidelines.xhtml

review's inclusion criteria. Few trials reported side effects or measures of participant satisfaction or wellbeing.

Trials generally included women with GDM diagnosed following a 75g or 100g oral glucose tolerance test (OGTT) using a variety of international [33,34,35] and locally [36,37] recommended thresholds, though some included women with 'mild or borderline' GDM (positive OGCT, negative OGTT) and others included women with 'impaired glucose tolerance' (IGT), current diagnostic criteria[12,38] however may now consider these women as having GDM rather than a separate and milder condition.

Quality –Risk of bias assessment

Overall, reporting of, and many aspects of trial quality, was poor with the result that risk of bias was generally unclear or high (Supplementary Table 1). The randomisation procedure and group allocation was rarely described, although all trials reported that participants were 'randomised'. Blinding of participants, medical staff and outcome assessors was generally not reported, but as most trials include some additional intervention above routine care such as diet advice or a pharmacological treatment, it is probable that participants and most clinicians could not be blinded, though outcome assessment could have been. Most trials had reasonably complete outcome data and loss to follow-up was low, though for some trials analysis was not conducted on an intention to treat basis (so the analysis did not include all women randomised). Selective reporting was assessed as minimal as the majority of trials presented results for all pre-specified outcomes (the possibility that some trials collected data on outcomes, but did not report them cannot be ruled out however).

Generally, women were eligible for inclusion in trials evaluating pharmacological treatments if they were unable to achieve adequate glycaemic control with dietary and lifestyle management. Therefore there is the possibility that those included may have had more severe or refractory hyperglycaemia or may adhere less well to lifestyle interventions than those women who did not

For peer review only - http://bmjopen.bmj.com/site/about/guidelines.xhtml

require pharmacological treatments to control hyperglycaemia. The specific criteria for the addition of supplemental insulin in trials were often not reported, though some trials did report that supplemental insulin was prescribed if 'glycaemic control was not achieved by participants'. It is probable that thresholds for what is defined as 'good' control differed between trial centres (if multi-site) and trials.

Packages of care and dietary modification trials

Twelve trials evaluated a package of care (a combination of treatments starting with dietary modification and/or exercise and/or monitoring and/or supplemental pharmacological treatments) (Table 1)[27-30,39-46] compared to routine care. Data from these 12 trials are combined in at least one meta-analysis (Figure 2a).

Seven trials[31,32,47-52] evaluated a variety of dietary modifications and compared them to other dietary modifications (Table 1). The composition of each dietary modification was generally well reported, however the interventions and comparisons were too diverse to allow pooling of data. There was no evidence that one type of dietary modification was superior over another, though trials included few women (Figure 2a). None of these seven trials reported side effects or quality of life measures.

The composition of the dietary modification was poorly reported in the 'packages of care' trials (the 12 trials included in the meta-analyses). Overall (in all packages of care and dietary modification trials), 10 out of 19 trials reported that insulin was provided if required, in one trial insulin was only provided if needed in the intervention group and for the remainder it was unclear or not reported if supplemental insulin was provided. The screening and diagnostic tests, criteria and glucose thresholds used to define GDM (and included/exclude women in the trials) varied across the trials

For peer review only - http://bmjopen.bmj.com/site/about/guidelines.xhtml

(Table 1). For the meta-analysis the varying forms of dietary modification and/or pharmacological treatment use was not examined.

Packages of care (starting with dietary modification and possibly including monitoring and pharmacological interventions) reduced the risk of shoulder dystocia by 60%, LGA and macrosomia by around 50%, pre-eclampsia by 20% and the incidence of Caesarean section by 10% compared to routine care (Figure 2a) though for pre-eclampsia and Caesarean section the confidence intervals included the null value. BW was reduced by approximately 110g in the packages of care compared to routine care group (Figure 2a). The degree of heterogeneity (I^2) varied by outcome from 0% to 77%. No ‘packages of care trial’ reported side effects; two trials reported quality of life scores[41,42] indicating higher (better) quality of life scores for women in the intervention compared to the routine care group.

Trials comparing metformin with insulin

Eleven trials compared metformin with Insulin (Table 2).[24,25,37,53-60] However most trials reported supplemental insulin use in the metformin group with the exception of two trials.[25,59] The risk of most outcomes, including LGA, macrosomia, NICU admission, neonatal hypoglycaemia, pre-eclampsia, PIH and induction of labour (IOL), was lower in those randomised to metformin rather than insulin; instrumental delivery was greater in those randomised to insulin (Figure 2b). Birthweight, gestational age and Apgar score as continuous measurements did not differ notably between the two treatments (Figure 2b). Six trials reported the proportion of women with metformin associated gastrointestinal upset (between 4% to 46%).[24,53-55,58,60] No trial reported quality of life measures.

For peer review only - http://bmjopen.bmj.com/site/about/guidelines.xhtml

Trials comparing glibenclamide (glyburide) with insulin

Nine trials compared glibenclamide with insulin (Table 3).[26,61-68] Figure 2c shows the relative risks of dichotomous outcomes, suggesting insulin may be relatively more effective than glibenclamide in reducing the risk of several adverse outcomes, confidence intervals are wide and include the null value however. There was no difference between insulin and glibenclamide for continuous outcomes (Figure 2c). One trial reported that glibenclamide was associated with side effects in 3/48 (6%) of women.[66] No trial reported quality of life measures .

Trials comparing glibenclamide (glyburide) with metformin

Only three trials were identified that directly compared glibenclamide with metformin and these were relatively small trials including between 149 and 200 women (Table 4).[69-71] Figure 2d shows the risk of dichotomous and continuous outcomes respectively. These suggest metformin is more effective at reducing risk of LGA and possibly macrosomia. However, for several of the outcomes (for example LGA) only data from one of these trials is available, it is therefore not possible to make robust conclusions about the relative benefits of metformin and glibenclamide from these direct comparisons. No trials reported side effects or quality of life measures.

Network meta-analysis comparing glibenclamide (glyburide), insulin and metformin

Table 5 the estimated probability of a treatment being the most effective at reducing the risk of each dichotomous outcome. Only dichotomous outcomes reported in at least two glibenclamide trials (either in comparison to insulin or metformin) were included in these analyses to ensure there were sufficient trials (and participants) included. When all three treatments are jointly compared, these analyses suggest that, for all outcomes, with the exception of Caesarean section, metformin is most likely to be the most effective treatment, with its probability of being most effective in reducing risk being 96.3, 94.0%, 92.8%, 84.0% and 61.2% respectively for neonatal hypoglycaemia, macrosomia,

For peer review only - http://bmjopen.bmj.com/site/about/guidelines.xhtml

For peer review only

LGA, pre-eclampsia and admission to NICU (the probability of being most effective for reducing risk of Caesarean section was 9.7% for metformin, glibenclamide was most likely to be most effective at reducing the risk of Caesarean section (79.9%)). Supplementary Figure 3 shows all two way comparisons between the treatments (e.g. metformin vs insulin). These confirm the direct comparisons described above, in suggesting that metformin is more effective than insulin or gliblenclamide at reducing the majority of adverse outcomes, however many of these comparisons are based on small numbers and have wide confidence intervals that sometimes include the null value.

Discussion

The key finding of our review is that, despite understanding of hyperglycaemia/GDM and its relationship to adverse perinatal outcomes having existed for at least seven decades,[72] and 42 RCTs completed on its treatment; trials are still being conducted that are of limited size and of poor quality (with subsequent unclear or high risk of bias). Given the changing characteristics of the population and the lower fasting diagnostic threshold (compared to previous criteria[34]) recommended by the IADPSG[38] and UK NICE,[12] it is important to understand how treatments affect outcomes for these women. Trials do not always report GDM diagnostic criteria clearly and this is important considering the potential influence on GDM population size and the magnitude of effect.[12,38] Our detailed review, including only evidence from RCTs, provides some support for a 'step up approach' in the treatment of hyperglycaemia, from dietary interventions, through addition of metformin (in preference to glibenclamide (glyburide)) through to addition of insulin. Considering hyperglycaemia in pregnancy has various causes, using an integrated individual approach to its management, is likely to work best.

We have taken a pragmatic approach to evaluating the many trials examining treatment packages of care for women diagnosed with hyperglycaemia/GDM so that our results will be generalisable to

For peer review only - http://bmjopen.bmj.com/site/about/guidelines.xhtml

most clinical situations. Previous reviews have also suggested packages of care with a 'step up' approach are most effective.[1,3-5] The number of trials and women included in previous reviews varies. One of the most recent reviews had broadly similar inclusion criteria to ours, comparing any package of care for the treatment of GDM with no treatment (routine care) and included five trials with 2643 women.[3] Our review includes all these trials, plus a further seven (included in the meta-analysis) increasing the number of women to 4512 and indicating that RCTs in this area continue to be conducted, but not with the size or quality that allows us to have a robust evidence base for the treatment of GDM in a contemporary population. Pooled estimates are generally consistent across reviews irrespective of the number of trials included, because estimates are driven in all reviews by the two largest, which are also the highest quality trials, however these trials were conducted in populations using diagnostic criteria that would provide populations with more severe hyperglycaemia (and therefore the potential for a larger effect size).[41,44] For example, our analysis shows the risk of macrosomia is halved when a package of care is provided compared to routine care (11 trials, RR 0.49, 95% CI 0.39-0.62), confirming estimates from the most recent previous review (RR 0.50 95% CI 0.35-0.71).[3] These two large and well-conducted RCTs were published in 2005 and 2009,[41,44] and since then several smaller and poorer quality trials have been published. These two previous large well-conducted trials cannot provide precise estimates of effect on the wider range of adverse outcomes and for women diagnosed using more recently recommended criteria. Hence, we feel it is important to place a moratorium on further small RCTs in this area and that funders should consider commissioning a multi-centre large-scale RCT with adequate power to determine the effect and cost-effectiveness of different packages of care on adverse outcomes in women with GDM.

The evidence to support metformin use, though encouraging has certain weaknesses. Firstly although there is a general 'trend' in favour of metformin use over insulin and glibenclamide (glyburide), confidence intervals are wide, in both the direct and network meta-analysis comparing each two-way treatment effect. Secondly the reporting of trial methods was generally poor with

For peer review only - http://bmjopen.bmj.com/site/about/guidelines.xhtml

'unclear or high risk of bias' and many trials included relatively few women and reported few outcomes. Thirdly, in most trials directly comparing metformin with insulin, women receiving metformin were also given supplemental insulin 'if required'; in one of the largest trials this equated to 46% of the metformin group.[54] Therefore our results more appropriately relate to metformin's greater effectiveness as a first-line treatment for GDM rather than a standalone treatment compared to insulin.

In addition to being an effective first-line pharmacological treatment for GDM, metformin may also be preferred by women as it is administered orally and can be stored at room temperature, compared to insulin that requires subcutaneous injection and refrigerated storage. Metformin is sometimes associated with gastrointestinal upset, which may affect compliance and quality of life.

Few trials have reported side effects or measures of participant satisfaction or wellbeing, all important outcomes that have the potential to impact health and therefore should be evaluated. Recent guidance[12,38] recommends lower glucose thresholds compared to those previously recommended to diagnose GDM[33,34] (and used in the included trials). Therefore it is possible that a greater proportion of women diagnosed with GDM will require only diet modification or less 'intensive' management compared to those previously diagnosed with GDM, because their hyperglycaemia is less severe. There is a continuum of increasing risk of adverse outcomes across the spectrum of glucose however[73,74] therefore interventions to reduce hyperglycaemia even at lower glucose levels are likely to improve outcomes, but this needs confirming by large well-designed RCTs.

For peer review only - http://bmjopen.bmj.com/site/about/guidelines.xhtml

Strengths and limitations

This systematic review and meta-analysis includes a large number of trials with varied populations, and examines the effectiveness of treatment packages and diets as well as individual pharmacological treatments for reducing the risk of adverse perinatal outcomes.

For some comparisons, trials and numbers of women were few, as were outcomes reported. Trial quality was generally poor with subsequent high or unclear risk of bias. GDM diagnostic criteria varied across trials and recently recommended thresholds are lower now compared to when most included trials were conducted.

Lower glucose threshold criteria recommended by the International Association of Diabetes and Pregnancy Study Groups[38] and subsequently endorsed by the World Health Organization[75] aim to identify offspring at risk of obesity through its association with LGA (birth weight >90th percentile), cord C-peptide >90th percentile and percentage body fat >90th percentile. However there are no trials that have used these criteria and the classification of less severe hyperglycaemia when lower glucose thresholds are used to diagnose GDM may reduce the magnitude of the effect of interventions, compared to those reported by earlier trials using higher glucose thresholds. There has also been no longer-term follow up conducted to evaluate the treatment of GDM and the effects on risk of offspring obesity. Importantly, few of the trials that we reviewed had reported side effects or measures of participant satisfaction or wellbeing.

Implications for practice

This review provides reassurance that a package of care where a 'step up' approach of firstly providing dietary and lifestyle advice, then adding supplementary metformin or insulin if glucose levels are not adequately controlled, is a reasonable and effective approach compared to providing just routine antenatal care, particularly with regards to reducing the risk of LGA. However, it has also

For peer review only - http://bmjopen.bmj.com/site/about/guidelines.xhtml

highlighted the general poor quality of recent small RCTs that do not improve the evidence base, but subject women with GDM to unnecessary ‘experimentation’ and are a cost to society.

Metformin seems to be an effective alternative to insulin, if diet modification inadequately controls hyperglycaemia, however supplemental insulin may be required in up to 50% of women.[54] There is a need to cease further small RCTs in this area and conduct large well-designed RCTs that clarify the most effective treatment across a range of outcomes, including those that are likely to be important to women such as quality of life measurements and those identified by the Cochrane Pregnancy and Childbirth Group (CPCG) as being essential for trials and reviews of diabetes in pregnancy. These should be incorporated into current diagnostic criteria and ideally look at longer-term outcomes in mothers and offspring.

Acknowledgements

Thank you to Julie Glanville and Mick Arber of the York Health Economics Consortium, University of York, UK, who carried out the searches.

Details of ethics approval

This study did not require ethical approval as the data used have been published previously, and hence are already in the public domain.

Contribution to authorship

DF, MS, DAL and TAS designed the study. MS wrote the statistical analysis plan. DF monitored the review process. DF, MS, MB, DAL, TAS, DT and FD interpreted the data, DF, MS, MB and SG assessed studies for inclusion. MS cleaned and analysed the data, DF wrote the draft paper. All authors have approved the final version. Diane Farrar is guarantor and takes responsibility for the content of this article.

For peer review only - http://bmjopen.bmj.com/site/about/guidelines.xhtml

Funding

This work was supported by the National Institute for Health Research (NIHR), Health Technology Assessment (HTA) programme, project number 11/99/02. DF holds a NIHR Post-doctoral Research Fellowship award (PD-2014-07-019). DAL works in a Unit that is supported by the University of Bristol and UK Medical Research Council ((MC_UU_12013/5) and she holds a NIHR Senior Investigator award (NF-SI-0611-10196).The views and opinions expressed therein are those of the authors and do not necessarily reflect those of the HTA, NIHR, MRC, UK National Health Service (NHS) or the Department of Health.

Data sharing

Extracted data are available upon request to the corresponding author

Competing interests: All authors have completed the ICMJE uniform disclosure form at www.icmje.org/coi_disclosure.pdf and declare: no support from any organisation for the submitted work; no financial relationships with any organisations that might have an interest in the submitted work in the previous three years; no other relationships or activities that could appear to have influenced the submitted work.

For peer review only - http://bmjopen.bmj.com/site/about/guidelines.xhtml

References

1. Alwan N, Tuffnell D, West J. Treatments for gestational diabetes. *Cochrane Database of Syst Rev*. 2009; Issue 3:CD003395.
2. Gui J, Liu Q, Feng L. Metformin vs insulin in the management of gestational diabetes: a meta-analysis. *PloS one*. 2013; 8(5): e64585.
3. Hartling L, Dryden DM, Guthrie A, Muise M, Vandermeer B, Donovan L. Benefits and harms of treating gestational diabetes mellitus: a systematic review and meta-analysis for the U.S. Preventive Services Task Force and the National Institutes of Health Office of Medical Applications of Research. *Ann Intern Med*. 2013; 159(2): 123-9.
4. Falavigna M, Prestes I, Schmidt MI, Duncan BB, Colagiuri S, Roglic G. Impact of gestational diabetes mellitus screening strategies on perinatal outcomes: a simulation study. *Diabetes Res Clin Pract*. 2012; 99(3): 358-65.
5. Horvath K, Koch K, Jeitler K, Matyas E, Ralf Bender R, Hilda Bastian H, et al. Effects of treatment in women with gestational diabetes mellitus: systematic review and meta-analysis. *BMJ*. 2010; 340: 1395-413.
6. Balsells M, García-Patterson A, Solà I, Roqué M, Gich I, Corcoy R. Glibenclamide, metformin, and insulin for the treatment of gestational diabetes: a systematic review and meta-analysis. BMJ [Journal Article] 2015 2015-01-21 16:06:23 [cited 350; Available from: http://www.bmj.com/bmj/350/bmj.h102.full.pdf
7. Han S, Crowther CA, Middleton P, Heatley E. Different types of dietary advice for women with gestational diabetes mellitus. *Cochrane Database Syst Rev*. 2013; (3).
8. Jiang Y-F, Chen X-Y, Ding T, Wang X-F, Zhu Z-N, Su S-W. Comparative efficacy and safety of OADs in management of GDM: network meta-analysis of randomized controlled trials. *J Clin Endocrinol Meteb*. 2015; 100(5): 2071-80.
9. Amin M, Suksomboon N, Poolsup N, Malik O. Comparison of glyburide with metformin in treating gestational diabetes mellitus: a systematic review and meta-analysis. *Clin Drug Investig*. 2015; 35(6): 343-51.
10. Kitwitee P, Limwattananon S, Limwattananon C, Waleekachonlert O, Ratanachotpanich T, Phimphilai M, et al. Metformin for the treatment of gestational diabetes: An updated meta-analysis. *Diabetes Res Clin Pract*. 2015; 109(3): 521-32.
11. Zhao L-P, Sheng X-Y, Zhou S, Yang T, Ma L-Y, Zhou Y, et al. Metformin versus insulin for gestational diabetes mellitus: a meta-analysis. *Br J Clin Pharmacol*. 2015; 80(5): 1224-34.
12. National Institute for Health and Care Excellence. Diabetes in pregnancy: management of diabetes and its complications from preconception to the postnatal period. *National collaborating centre for Women's and Children's Health*. 2015.
13. Higgins JPT, Green S, editors. Cochrane Handbook for Systematic Reviews of Interventions Version 5.1.0 [updated March 2011]. *The Cochrane Collaboration*. 2011; Available from www.cochrane-handbook.org.
14. Centre for Reviews and Dissemination. Systematic reviews: CRD's guidance for undertaking a systematic review York: CRD, University of York

2009.
15. Preferred Reporting Items for Systematic Reviews and Meta-Analyses. PRISMA. *http://wwwprisma-statementorg*. Last accessed June 2015.
16. Farrar D, Fairley L, Tuffnell D, Wright J, Sheldon T, Bland M, et al. A clinical and economic evaluation of screening and diagnostic tests to identify and treat women with gestational diabetes: association between maternal risk factors, glucose levels, and adverse outcomes. *HTA*. 2013; http://www.nets.nihr.ac.uk/projects/hta/119902.
17. Sakala C, Gyte G, Henderson S, Neilson JP, Horey D. Consumer-Professional Partnership to Improve Research: The Experience of the Cochrane Collaboration's Pregnancy and Childbirth Group. *Birth*. 2001; 28(2): 133-7.

For peer review only - http://bmjopen.bmj.com/site/about/guidelines.xhtml

18. The Cochrane Collaboration. The Cochrane Collaboration's tool for assessing risk of bias. *http://ohgcochraneorg/sites/ohgcochraneorg/files/uploads/Risk%20of%20bias%20assessment%20toolpdf*. last accessed Jan 2014.
19. DerSimonian R, Laird N. Meta-analysis in clinical trials. *Control Clin Trials*. 1986; 7(3): 177-88.
20. Higgins JPT, Thompson SG. Quantifying heterogeneity in a meta-analysis. *Stats Med*. 2002; 21(11): 1539-58.
21. Bafeta A, Trinquart L, Seror R, Ravaud P. Reporting of results from network meta-analyses: methodological systematic review. *BMJ*. 2014; 348.
22. Lu G, Ades AE. Combination of direct and indirect evidence in mixed treatment comparisons. *Statis Med*. 2004; 23(20): 3105-24.
23. openbugs. http://www.openbugs.net/w/FrontPage. last accessed January 2015.
24. Ainuddin J, Karim N, Hasan AA, Naqv iSA. Metformin versus insulin treatment in gestational diabetes in pregnancy in a developing country. A randomized control trial. *Diab Res Clin Prac*. 2014.
25. Zinnat ANS, Shareen S, Rahman S. Can metformin be used in place of insulin for the treatment of GDM for low resource countries? *BJOG*. 2013; 120: 35.
26. Mirzamoradi M, Bakhtiyari M, Kimiaee P, Hosseini-Najarkolaei A, Mansournia MA. Investigating the effects of treatment based on single high blood glucose in gestational diabetes screening on maternal and neonatal complications. Arch Gynecol Obst; 2015. p. 687-95.
27. Deveer R, Deveer M, Akbaba E, Engin-Ustun Y, Aydogan P, Celikkaya H, et al. The effect of diet on pregnancy outcomes among pregnant with abnormal glucose challenge test. *Eur Rev Med Pharmacol Sci*. 2013; 17(9): 1258-61.
28. Fadl HE, Gardefors S, Hjertberg R, Nord E, Persson B, Schwarcz E, et al. Randomized controlled study in pregnancy on treatment of marked hyperglycemia that is short of overt diabetes. *Acta Obstet Gynecol Scand*. 2015; 94(11): 1181-7.
29. Yang X, Hsu-Hage B, Dong L, Shao P, Wang H, Tian H, et al. Intensive Diabetes Management May Improve Pregnancy. *Diabetes Care*. 2003; 26(1): 254.
30. Yang X, Tian H, Zhang F, Zhang C, Li Y, Leng J, et al. A randomised translational trial of lifestyle intervention using a 3-tier shared care approach on pregnancy outcomes in Chinese women with gestational diabetes mellitus but without diabetes. J Transl Med; 2014.
31. Ma W-J, Huang Z-H, Huang B-X, Qi B-H, Zhang Y-J, Xiao B-X, et al. Intensive low-glycaemic-load dietary intervention for the management of glycaemia and serum lipids among women with gestational diabetes: a randomized control trial. *Public Health Nutr*. 2015; 18(8): 1506-13.
32. Yao J, Cong L, Zhu B, Wang T. Effect of dietary approaches to stop hypertension diet plan on pregnancy outcome patients with gestational diabetes mellitus. *Bangladesh J Pharmacol*. 2015; 10(4): 732-8.
33. American College of Obstetricians and Gynecologists. Practice Bulletin Clinical Management Guidelines for Obstetricians - Gynecologists. *Obstet Gynecol*. 2013; 122(2): 406- 16.
34. World Health Organization. Definition, diagnosis and classification of diabetes mellitus and its complications. Report of a WHO consultation. Part 1: diagnosis and classification of diabetes mellitus. Geneva: WHO; 1999.
35. American Diabetes Association. Standards of Medical Care in Diabetes - 2014. *Diabetes Care* 2014; 37(Supp 1): S14-80.
36. Ijäs H, Vääräsmäki M, Morin-Papunen L, Keravuo R, Ebeling T, Saarela T, et al. Metformin should be considered in the treatment of gestational diabetes: a prospective randomised study. *BJOG*. 2011; 118(7): 880-5.
37. Tertti K, Ekblad U, Koskinen P, Vahlberg T, Ronnemaa T. Metformin vs. insulin in gestational diabetes. A randomized study characterizing metformin patients needing additional insulin. *Diabetes Obes Metab*. 2013; 15(3): 246-51.
38. International Association of Diabetes and Pregnancy Study Groups Consensus panel. International Association of Diabetes and Pregnancy Study Groups Recommendations on the Diagnosis and Classification of Hyperglycemia in Pregnancy. *Diabetes Care*. 2010; 33(7): 676-82.

For peer review only - http://bmjopen.bmj.com/site/about/guidelines.xhtml

39. Bevier WC, Fischer R, Jovanovic L. Treatment of women with an abnormal glucose challenge test (but a normal oral glucose tolerance test) decreases the prevalence of macrosomia. Am J Perinatol; 1999. p. 269-75.
40. Bonomo M, Corica D, Mion E, Goncalves D, Motta G, Merati R, et al. Evaluating the therapeutic approach in pregnancies complicated by borderline glucose intolerance: a randomized clinical trial. *Diabet Med*. 2005; 22(11): 1536-41.
41. Crowther CA, Hiller JE, Moss JR, McPhee AJ, Jeffries WS, Robinson JS. Effect of treatment of gestational diabetes mellitus on pregnancy outcomes. *N Engl J Med*. 2005; 352(24): 2477-86.
42. Elnour AA, Mugammar IT, Jaber T, Revel T, McElnay JC. Pharmaceutical care of patients with gestational diabetes mellitus. J evaluat clin prac; 2008. p. 131-40.
43. Garner P, Okun N, Keely E, Wells G, Perkins S, Sylvain J, et al. A randomized controlled trial of strict glycemic control and tertiary level obstetric care versus routine obstetric care in the management of gestational diabetes: A pilot study. *Am J Obstet Gynecol*. 1997; 177(1): 190-5.
44. Landon MB, Spong CY, Thom E, Carpenter MW, Ramin SM, Casey B, et al. A multicenter, randomized trial of treatment for mild gestational diabetes. *N Engl J Med*. 2009; 361: 1339-48.
45. Li DF, Wong VC, O'Hoy KM, Yeung CY, Ma HK. Is treatment needed for mild impairment of glucose tolerance in pregnancy? A randomized controlled trial. BJOG; 1987. p. 851-4.
46. O'Sullivan JB, Mahan CM. Insulin treatment and high risk groups. *Diabetes Care*. 1980; 3(3): 482-5.
47. Asemi Z, Samimi M, Tabassi Z, Esmaillzadeh A. The effect of DASH diet on pregnancy outcomes in gestational diabetes: a randomized controlled clinical trial. *Eur J Clin Nutr*. 2014; 68(4): 490-5.
48. Cao X, Wang Z, Yang C, Mo X, Xiu L, Li Y, et al. Comprehensive intensive therapy for Chinese gestational diabetes benefits both newborns and mothers. *Diabetes Technol Ther*. 2012; 14(11).
49. Cypryk K, Kaminska P, Kosinski M, Petynska-Marczewska M, Lewinski A. A comparison of the effectiveness, tolerability and safety of high and low carbohydrate diets in women with gestational diabetes. Endokryno Pol; 2007. p. 314-9.
50. Louie JCY, Markovic TP, Perera N, Foote D, Petocz P, Ross GP, et al. A randomized controlled trial investigating the effects of a low-glycemic index diet on pregnancy outcomes in gestational diabetes mellitus. *Diabetes Care*. 2011; 34(11): 2341-6.
51. Moreno-Castilla C, Hernandez M, Bergua M, Alvarez MC, Arce MA, Rodriguez K, et al. Low-Carbohydrate Diet for the Treatment of Gestational Diabetes Mellitus: A randomized controlled trial. *Diabetes Care*. 2013; 36(8): 2233-8.
52. Rae A, Bond D, Evans S, North F, Roberman B, Walters B. A randomised controlled trial of dietary energy restriction in the management of obese women with gestational diabetes. Aust N Z J Obstet Gynaecol; 2000. p. 416-22.
53. Spaulonci CP, Bernardes LS, Trindade TC, Zugaib M, Francisco RPV. Randomized trial of metformin vs insulin in the management of gestational diabetes. *Am J Obstet Gynecol*. 2013; 209(1): 34e1-e7.
54. Rowan JA, Hague WM, Gao W, Battin MR, Moore MP. Metformin versus Insulin for the Treatment of Gestational Diabetes. *N Engl J Med*. 2008; 358(19): 2003-15.
55. Niromanesh S, Alavi A, Sharbaf FR, Amjadi N, Moosavi S, Akbari S. Metformin compared with insulin in the management of gestational diabetes mellitus: A randomized clinical trial. *Diabetes Res Clin Pract*. 2012; 98(3): 422-9.
56. Moore LE, Briery CM, Clokey D, Martin RW, Williford NJ, Bofill JA, et al. Metformin and insulin in the management of gestational diabetes mellitus: Preliminary results of a comparison. *J Rep Med Obstet Gynecol*. 2007; 52(11): 1011-5.
57. Mesdaghinia E, Samimi M, Homaei Z, Saberi F, Moosavi SGA, Yaribakht M. Comparison of newborn outcomes in women with gestational diabetes mellitus treated with metformin or insulin: a randomised blinded trial. *Int J Prev Med*. 2013; 4(3): 327-33.

For peer review only - http://bmjopen.bmj.com/site/about/guidelines.xhtml

58. Ijäs H, Vääräsmäki M, Morin-Papunen L, Keravuo R, Ebeling T, Saarela T, et al. Metformin should be considered in the treatment of gestational diabetes: a prospective randomised study. BJOG; 2010. p. 880-5.
59. Hague W, Davoren P, Oliver J, Rowan J. Contraindications to use of metformin. Metformin may be useful in gestational diabetes. *BMJ*. 2003; 326: 762.
60. Hassan JA, Karim N, Sheikh Z. Metformin prevents macrosomia and neonatal morbidity in gestational diabetes. *Pak J Med Sci*. 2012; 28(3): 384-9.
61. Anjalakshi C, Balaji V, Balaji MS, Seshiah V. A prospective study comparing insulin and glibenclamide in gestational diabetes mellitus in Asian Indian women. *Diabetes Res Clin Pract*. 2007; 76(3): 474-5.
62. Bertini AM, Silva JC, Taborda W, Becker F, Lemos Bebber FR, Zucco Viesi JM, et al. Perinatal outcomes and the use of oral hypoglycemic agents. J perinat med; 2005. p. 519-23.
63. Lain KY, Garabedian MJ, Daftary A, Jeyabalan A. Neonatal adiposity following maternal treatment of gestational diabetes with glyburide compared with insulin. *Am J Obstet Gynecol*. 2009; 200(5): 501.e1-.e6.
64. Langer O, Conway DL, Berkus MD, Xenakis EM-J, Gonzales O. A Comparison of Glyburide and Insulin in Women with Gestational Diabetes Mellitus. *N Engl J Med*. 2000; 343(16): 1134-8.
65. Mukhopadhyay P, Bag TS, Kyal A, Saha DP, Khalid N. Oral hypoglycemic glibenclamide: Can it be a substitute to insulin in the management of gestational diabetes mellitus? a comparative study. *J South Asian Feder Obstet Gynae*. 2012; 4(1): 28-31.
66. Ogunyemi D, Jesse M, Davidson M. Comparison of glyburide versus insulin in management of gestational diabetes mellitus. Endo prac; 2007. p. 427-8.
67. Silva J, Bertini A, Taborda W, F. B, Bebber F, Aquim G, et al. Glibenclamide in the Treatment for Gestational Diabetes Mellitus in a Compared Study to Insulin. *Arq Bras Endocrinol Metab*. 2007; 51(4): 541-6.
68. Tempe A, Mayanglambam RD. Glyburide as treatment option for gestational diabetes mellitus. *J Obstet Gynaecol Res*. 2013; 39(6): 1147-52.
69. Moore LE, Clokey D, Rappaport VJ, Curet LB. Metformin compared with glyburide in gestational diabetes: a randomized controlled trial. Obst Gynecol; 2010. p. 55-9.
70. Silva JC, Pacheco C, Bizato J, Souza BV, Ribeiro TE, Bertini AM. Metformin compared with glyburide for the management of gestational diabetes. Int J Gynae Obst; 2010. p. 37-40.
71. George A, Mathews JE, Sam D, Beck M, Benjamin SJ, Abraham A, et al. Comparison of neonatal outcomes in women with gestational diabetes with moderate hyperglycaemia on metformin or glibenclamide - A randomised controlled trial. Aust N Z J Obstet Gynaecol; 2015. p. 47-52.
72. Lawlor DA. The Society for Social Medicine John Pemberton Lecture 2011. Developmental overnutrition—an old hypothesis with new importance? *Int J Epidemiol*. 2013; 42 (1): 7-29.
73. Metzger BE LL, Dyer AR, et al. HAPO Study Cooperative,. Hyperglycemia and Adverse Pregnancy Outcomes. *N Engl J Med*. 2008; 358: 1991-2002.
74. Farrar D, Fairley L, Santorelli G, Tuffnell D, Sheldon TA, Wright J, et al. Association between hyperglycaemia and adverse perinatal outcomes in south Asian and white British women: analysis of data from the Born in Bradford cohort. *Lancet Diabetes Endocrinol*. 2015; 3(10): 795-804.
75. World Health Organization. Diagnostic criteria and classification of hyperglycaemia first detected in pregnancy. Geneva: WHO; 2013.
76. O'Sullivan JB, Gellis SS, Dandrow RV, Tenney BO. The Potential Diabetic and Her Treatment in Pregnancy. *Obstet Gynecol*. 1966; 27(5): 683-9.
77. Silva JC, Fachin DRlRN, Coral ML, Bertini AM. Perinatal impact of the use of metformin and glyburide for the treatment of gestational diabetes mellitus. *J Perinat Med*. 2012; 40(3): 225-8.

For peer review only - http://bmjopen.bmj.com/site/about/guidelines.xhtml

Figure 1: Search process

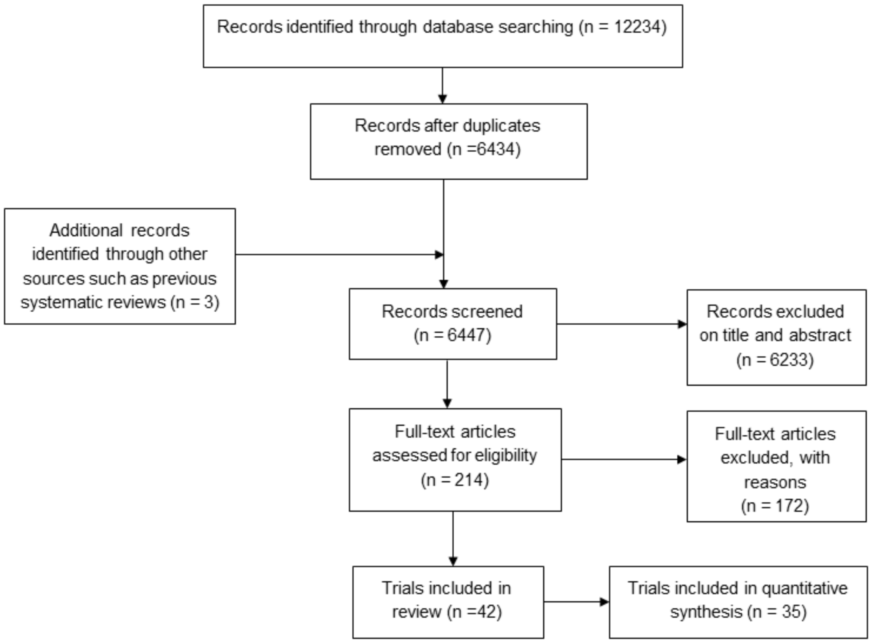

For peer review only - http://bmjopen.bmj.com/site/about/guidelines.xhtml

Figure 2: Forest plots for treatment comparisons and perinatal outcomes

2a: Packages of care (starting with dietary modification) versus routine care: dichotomous and continuous outcomes

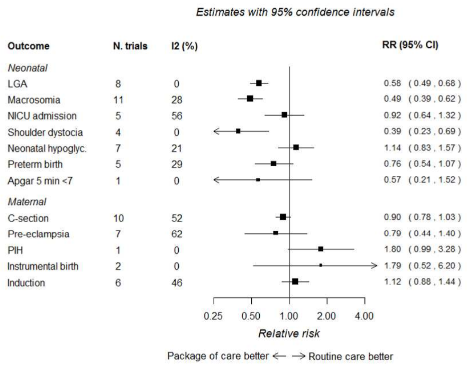

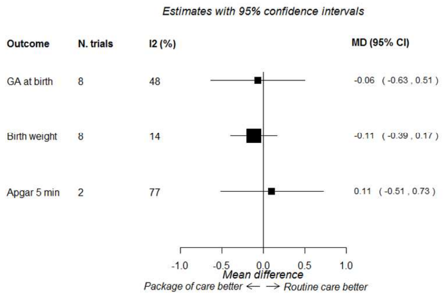

Figure 2b: Metformin versus insulin: dichotomous and continuous outcomes

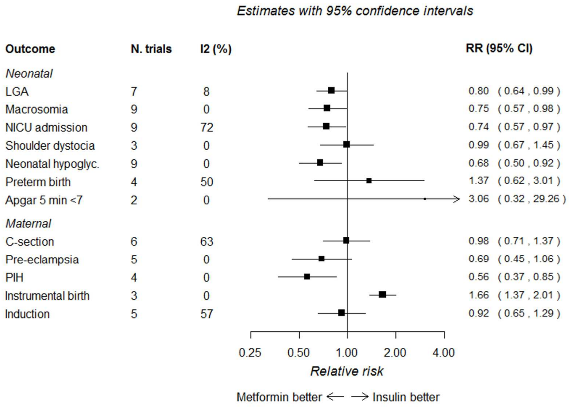

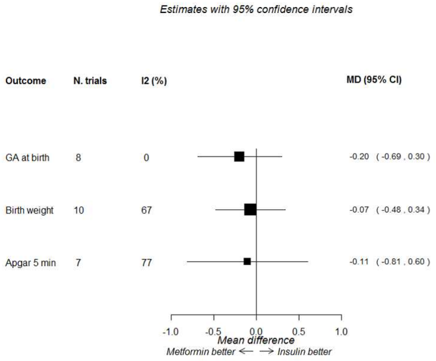

For peer review only - http://bmjopen.bmj.com/site/about/guidelines.xhtml

Figure 2c: Glibenclamide versus insulin: dichotomous and continuous outcomes

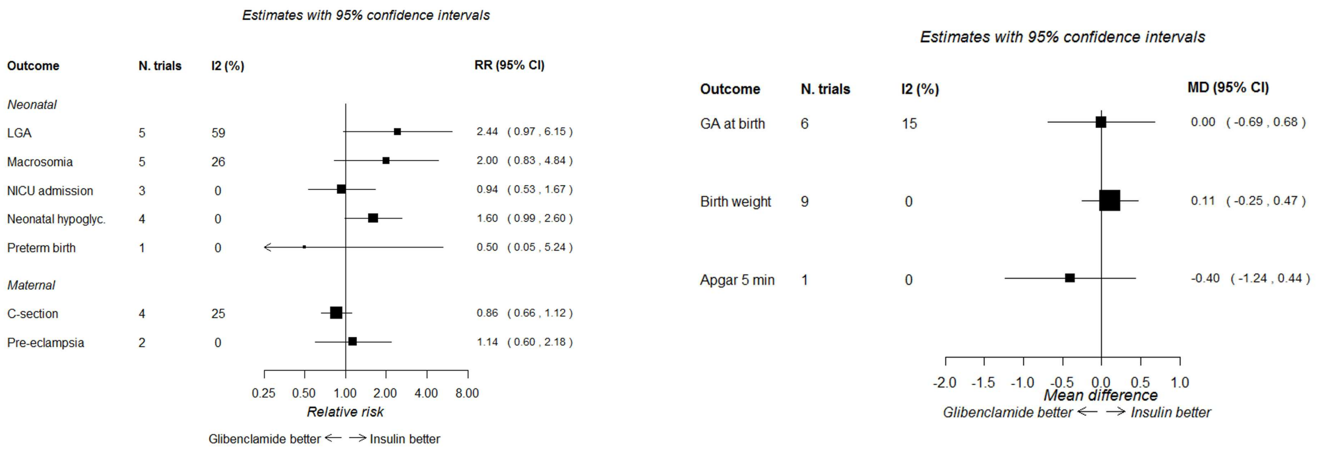

Figure 2d: Glibenclamide versus metformin: dichotomous and continuous outcomes

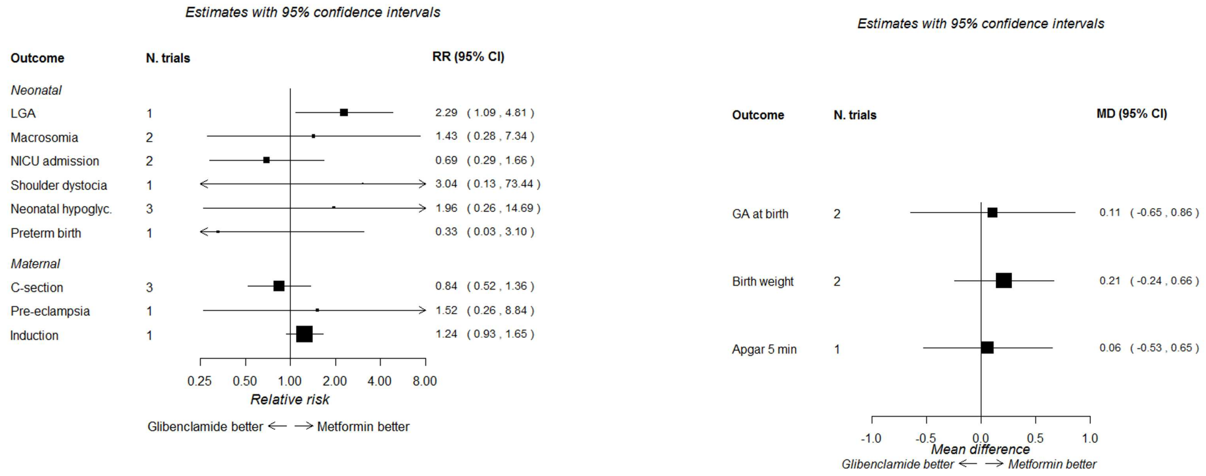

For peer review only - http://bmjopen.bmj.com/site/about/guidelines.xhtml

Table 1: Trials comparing a package of care starting with dietary modification to routine care and trials comparing a dietary modification with another dietary modification

First author	Year	Location	Number	Screening strategy used to determine need for diagnostic test	Diagnostic test and glucose thresholds used to diagnose GDM (mmol/L)	Intervention group	Control group	Insulin use in diet group	In meta-analyses	Meta-analysis outcome
Trials comparing a package of care (starting with dietary modification) to routine care										
Bevier[39]	1999	USA	103	50g OGCT >7.8	Positive OGCT, negative 100g OGTT, levels not reported	Dietary counselling and home monitoring	Routine care	If needed	yes	Apgar 5 min, BW, C-section, GA at birth, induction, instrumental birth, macrosomia, pre-eclampsia, shoulder dystocia
Bonomo[40]	2005	Italy	300	Risk factors and 50g OGCT	Positive OGCT $\geq$7.8, negative 100g OGTT 'C&C criteria'	Dietary advice and monitoring	Routine care	Not reported	yes	Apgar 5 min, BW, C-section, GA at birth, LGA, macrosomia, NN hypoglycaemia, NICU admission,

For peer review only - http://bmjopen.bmj.com/site/about/guidelines.xhtml

Crowther[41]	2005	UK / Australia	1000	Risk factors or 50g OGCT	75g OGTT fasting <7.8 and 2-hr >7.8 and < 11.1	Individualised dietary advice, monitoring & pharmacological treatments	Routine care	If needed	yes	Apgar 5 min <7, BW, C-sectionGA at birth, induction, macrosomia, NN hypoglycaemia, NICU admission, pre-eclampsia, shoulder dystocia
Deveer[27]	2013	Turkey	100	Universal 50g OGCT >7.8 and <10.0	Positive OGCT, negative 100g OGTT fasting <5.3 1-hr <10.0, 2-hr <8.8 and 3-hr <7.8	Calorie diet	Routine care	Not reported	yes	BW, C-section, gest age at birth, LGA, macrosomia, NICU admission, pre-eclampsia, preterm birth
Elnour[42]	2006	UAE	180	Not reported	100g OGTT, 'C&C criteria'	Diet education, exercise, monitoring & pharmacological treatments	Routine care	If needed	yes	C-section, LGA, macrosomia, NN hypoglycaemia, NICU admission, pre-eclampsia, preterm birth, shoulder dystocia

For peer review only

Fadl[28]	2015	Sweden	66	Risk factors	75g OGTT <7.0, ≥10.0 <12.2	Diet education, exercise, monitoring & pharmacological treatments	Routine care	If needed in intervention group only	yes	BW, C-section, LGA, GA at birth, macrosomia, pre-eclampsia, instrumental birth, induction, NICU admission
Garner[43]	1997	Canada	299	75g OGCT >8.0	75g OGTT fasting >7.5 and 2-hr >9.6	Dietary counselling, restricted calorie intake, monitoring & insulin if required	Routine care	If needed	yes	BW, C-section, GA at birth, macrosomia, NN hypoglycaemia, pre-eclampsia, preterm birth, shoulder dystocia
Landon[44]	2009	USA	958	50g OGCT >7.5-<11.1	100g OGTT fasting <5.3, 2 or more 1-hr >8.6 or 2-hr >8.6	Individualised dietary advice, monitoring & insulin	Routine care	If needed	yes	BW, C-section, GA at birth, induction, macrosomia, NN hypoglycaemia, NICU admission, pre-eclampsia, preterm birth, shoulder dystocia

For peer review only - http://bmjopen.bmj.com/site/about/guidelines.xhtml

Li[45]	1987	Hong Kong	58	Risk factors	100g OGTT, two or more: fasting >5.8, 1 hr >10.6, 2-hr >9.2, 3-hr >8.1, then 75g OGTT fasting <8.0 or 2-hr <11.0	30-35g/kg carbohydrate diet and monitoring	Routine care	Not reported	yes	BW, C-section, GA at birth, induction, macrosomia,
O'Sullivan[76]	1966	USA	615	OGCT or risk factors	100g OGTT two or more fasting > 6.1, or 1-hr > 9.1 or 2-hr > 6.7 or 3-hr >6.1	Low calorie diabetic diet	Standard diabetic diet	Only in intervention group	yes	Macrosomia, preterm birth
Yang[29]	2003	China	150	Not reported	Not reported	'intensive' diabetes management	Routine care	If needed	yes	C-Section, shoulder dystocia
Yang[30]	2014	China	700		75g OGTT fasting 5.1, 1 hr 10.0, 2 hr 8.5	Individual & group dietary/physical intervention	Routine care	If needed	yes	BW, C-section, GA at birth, induction, macrosomia, NN hypoglycaemia, PIH, pre-eclampsia, preterm birth, shoulder dystocia

Trials comparing a dietary modification with another dietary modification

For peer review only - http://bmjopen.bmj.com/site/about/guidelines.xhtml

Asemi[47]	2014	Iran	52	50g OGCT	OGCT ≥7.8, 75g OGTT Fasting: >5.1, 1 hr ≥10.0, 2 hr ≥8.5	DASH diet[a]	Control diet	Women with GDM excluded, therefore insulin not required	no	-
Cypryk[49]	2007	Poland	30	Not reported	levels not reported only that the WHO criteria was used	High carbohydrate diet	Low carbohydrate diet	If needed	no	-
Louie[50]	2011	Australia	99	Not reported	75g OGTT≥5.5, 1-hr ≥10.0 or 2-h ≥8.0	Low GI diet	High fibre moderate GI diet	If needed	no	-
Ma[31]	2015	China	83	50g OGCT	75g OGTT≥5.8, 1-hr ≥10.6, 2-h ≥9.2 or 3-hr 8.1	Low glycaemia load diet	Usual diet	If needed[b]	no	-
Moreno-Castilla[51]	2013	Spain	152	50g OGCT ≥7.8	100g OGTT >5.8, 1 hr >10.6, 2-hr >9.2, 3-hr >8.1	Low carbohydrate diet	Control diet	If needed	no	-
Rae[52]	2000	Australia	124	Not reported	(glucose load not reported) OGTT fasting >5.4 or 2-hour > 7.9	Calorie restricted diet	Usual diet	If needed	no	-
Yao[32]	2015	China	33	50g OGCT fasting ≥5.8 'post-load' ≥7.8	100g OGTT fasting >5.3, 1 hr >10.0, 2-hr >8.6, 3-hr >7.8	DASH diet	Usual diet	If needed	no	-

[a]DASH diet = Dietary Approaches to Stop Hypertension
[b]women who required insulin were excluded from the trial's analyses

For peer review only

Table 2: Trials comparing metformin to insulin

First author	Year	Location	Number	Diagnostic test and glucose thresholds used to diagnose GDM	Screening strategy[a]	Meta-analysis outcome
Ainuddin[24]	2014	Pakistan	150	75g OGTT two or more; fasting 5.3, 1 hr 10.0, 2 hr 8.6	50g OGCT≥7.8	PIH, pre-eclampsia, GA at delivery, induction, C-section, LGA, NICU admission, neonatal hypoglycaemia
Hague[59]	2003	Australia	30	75g OGTT fasting >5.5 or 2-hr >8.0	Risk factors	BW, Pre-eclampsia, GA at birth, induction, C-section, macrosomia, hypoglycaemia
Hassan[60]	2012	Pakistan	150	75g OGTT 2 or more levels fasting >5.3, 1-hr >10.0 or 2-hr >8.6	50g OGCT ≥7.8	Apgar 5 min, GA at birth, induction, C-section, BW, macrosomia, hypoglycaemia, NICU admission
Ijas[58]	2010	Finland	100	75g OGTT fasting >5.3, 1-hr >11.0 or 2-hr >9.6	Risk-based	Apgar 5 min, BW, C-section, GA at birth, induction, instrumental birth, LGA,

For peer review only - http://bmjopen.bmj.com/site/about/guidelines.xhtml

						macrosomia, hypoglycaemia, NICU admission
Mesdaghinia[57]	2013	Iran	200	100g OGTT two or more; fasting >5.3 or 1-hr >10.0 or 2-hr >8.6 or 3-hr >7.8	50g OGCT–levels not reported	BW, macrosomia, LGA, hypoglycaemia, NICU admission, shoulder dystocia, 5 min Apgar <7, preterm birth
Moore[56]	2007	USA	63	100g OGTT two or more; fasting >5.8 or 1-hr >10.5 or 2-hr >9.1 or 3-hr >8.0	50g OGCT ≥7.8	Apgar 5 min, BW, macrosomia, hypoglycaemia, NICU admission
Niromanesh[55]	2012	Iran	160	100g OGTT two or more fasting >5.3, 1-hr >10.0, 2-hr, 3-hr >8.6 or 3-hr >7.8	50g OGCT ≥7.2	Apgar 5 min, pre-eclampsia, PIH GA at birth, induction, C-section, shoulder dystocia, BW macrosomia, LGA, NICU admission, hypoglycaemia, preterm birth
Rowan[54]	2008	Australia / NZ	751	75g OGTT fasting >5.5 or 2-hr >8.0	Risk factors	Apgar 5 min <7, BW, GA at birth, LGA, NICU admission, PIH, pre-eclampsia, preterm birth
Spaulonci[53]	2013	Brazil	94	75g or 100g OGTT fasting >5.3 or 1-hr >10.0 or 2-hr >8.0	No screening	GA at birth, BW, Apgar 5 min, macrosomia,

For peer review only

				and two or more fasting >5.3, 1-hr >10.0, 2-hr, 3-hr >8.6 or 3-hr >7.8 respectively		hypoglycaemia, pre-eclampsia, preterm birth, C-section
Tertti[37]	2013	Finland	217	75g OGTT both criteria: fasting ≥4.8, 1-h ≥10.0, 2-h ≥8.7 and fasting ≥5.3, ≥10.0 and ≥8.6 respectively	Risk factors	GA at birth, BW, Apgar at 5 min, induction, instrumental birth, C-section, LGA, macrosomia, preterm birth, PIH, pre-eclampsia, NICU admission, hypoglycaemia
Zinnat[25]	2013	Bangladesh	450	Not reported[d]	Not reported[d]	Macrosomia, shoulder dystocia, C-section, instrumental birth hypoglycaemia, NICU admission,

[a]It is assumed unless otherwise reported, that the screening strategy advocated by the criteria used was adhered to
[d]Conference abstract

For peer review only

Table 3: Trials comparing glibenclamide (glyburide) to insulin

First author	Year	Location	Number	Diagnostic test and glucose thresholds used to diagnose GDM	Screening strategy[a]	Outcome
Anjalakshi[61]	2007	India	23	75g OGTT 2-hr >7.8	Universal OGTT	BW
Bertini[62]	2005	Brazil	70	75g OGTT fasting >6.1 or 2-hr >7.8	Not reported	BW, C-section, Apgar 5 min, GA at birth, LGA
Lain[63]	2009	USA	99	100g OGTT 2 or more: fasting >5.3, 1-hr >8.6 or 2-hr >8.6	50g >7.5	BW, GA at birth, LGA, macrosomia
Langer[64]	2000	USA	404	100g OGTT fasting >5.3-<7.8	50g OGCT >7.3	BW, C-section, GA at birth, LGA, macrosomia, hypoglycaemia, NICU admission, pre-eclampsia
Mirzamoradi[26]	2015	Iran	96	Glucose load not reported; OGTT 2 or more: fasting >5.3, 1-hr >10.0, 2-hr >8.3	Universal OGTT	BW, C-section, GA at birth, NICU admission, hypoglycaemia, pre-eclampsia
Mukhopadhyay[65]	2012	India	60	75g OGTT 2-hr >7.8	No screening	BW, GA at birth, LGA, hypoglycaemia
Ogunyemi[66]	2007	USA	97	Not reported	Not reported	BW, C-section, GA at birth, hypoglycaemia,

For peer review only - http://bmjopen.bmj.com/site/about/guidelines.xhtml

Silva[67]	2007	Brazil	68	75g OGTT fasting >6.1 or 2-hr >7.8	No screening	BW, C-section, LGA, macrosomia,
Tempe[68]	2013	India	64	100g OGTT 2 or more: fasting >5.3, 1-hr >10.0, 2-hr >8.6 or 3-hr >7.8	50g OGCT >7.2	BW, GA birth, macrosomia, hypoglycaemia, NICU admission, pre-eclampsia, preterm birth

[a]It is assumed unless otherwise reported, that the screening strategy advocated by the criteria used was adhered to

For peer review only

Table 4: Trials comparing glibenclamide to metformin

First author	Year	Location	Number	Diagnostic test and thresholds used to diagnose GDM (mmol/L)	Screening strategy[a]	Outcome
George[71]	2015	India	159	100g OGTT 2 or more; fasting ≥5.3 or 1 hr ≥10.0 or 2-hr ≥8.6	Not reported	BW, GA at birth, macrosomia, hypoglycaemia
Moore[69]	2010	USA	149	100g OGTT 2 or more; fasting >5.3 or 2-hr >6.7	50g OGCT>7.2	BW, C-section, GA at birth, macrosomia, hypoglycaemia, NICU admission, pre-eclampsia, shoulder dystocia
Silva[77]	2012	Brazil	200	75g OGTT fasting >5.3 or 1-hr >10.0 or 2-hr >8.0	No screening	Apgar 5 min, BW, C-section, GA at birth, LGA, macrosomia, hypoglycaemia, NICU admission

For peer review only

For peer review only - http://bmjopen.bmj.com/site/about/guidelines.xhtml

Table 5: Estimated probability (%) of a treatment being the most effective in reducing the risk of a dichotomous outcome.

	Treatment		
	**Insulin**	**Metformin**	**Glibenclamide (Glyburide)**
**Outcome**			
LGA	7.1	92.8	0.1
Macrosomia	5.6	94.0	0.3
Neonatal intensive care admission	0.5	61.2	38.3
Neonatal hypoglycaemia	3.3	96.3	0.4
Caesarean section	10.4	9.7	79.9
Pre-eclampsia	4.8	84.0	11.2

For peer review only

Supplementary file 1: Search strategy

1 exp diabetes, gestational/ (8715)
2 (gestation$ adj4 diabet$).ti,ab. (10162)
3 gdm.ti,ab. (4203)
4 (glucose adj4 (pregnan$ or gestation$ or prenatal$ or antenatal$ or pre-natal$ or ante-natal$ or maternal$)).ti,ab. (3796)
5 or/1-4 (15126)
6 Glucose Intolerance/ (7142)
7 Glucose Tolerance Test/ (31300)
8 IGT.ti,ab. (4074)
9 ((impair$ or reduced) adj2 glucose).ti,ab. (19442)
10 (glucose adj (tolerance$ or intolerance$)).ti,ab. (40791)
11 (gtt or ogtt).ti,ab. (7907)
12 Prediabetic State/ (4763)
13 (prediabet$ or pre-diabet$).ti,ab. (6103)
14 exp Insulin Resistance/ (64450)
15 (metabolic syndrome$ or syndrome$ x or borderline diabet$).ti,ab. (37636)
16 or/6-15 (134039)
17 exp Pregnancy/ (795751)
18 (pregnan$ or gestation$ or prenatal$ or antenatal$ or pre-natal$ or ante-natal$ or maternal$).ti,ab. (639369)
19 or/17-18 (1008161)
20 16 and 19 (10229)
21 5 or 20 (20405)
22 randomized controlled trial.pt. (421926)
23 controlled clinical trial.pt. (91079)
24 random$.ti,ab. (841233)
25 placebo.ti,ab. (176519)
26 drug therapy.fs. (1876752)
27 trial.ti,ab. (430134)
28 groups.ab. (1574965)
29 or/22-28 (3970247)
30 21 and 29 (6337)
31 (2014$ or 2015$ or 2016$).ed,dc,dp,ep,vd,yr. (3346601)
32 30 and 31 (1671)
33 animals/ not humans/ (4235813)
34 32 not 33 (1555)

For peer review only

PRISMA 2009 Checklist

Section/topic	#	Checklist item	Reported on page #
**TITLE**			
Title	1	Identify the report as a systematic review, meta-analysis, or both.	1
**ABSTRACT**			
Structured summary	2	Provide a structured summary including, as applicable: background; objectives; data sources; study eligibility criteria, participants, and interventions; study appraisal and synthesis methods; results; limitations; conclusions and implications of key findings; systematic review registration number.	2-3
**INTRODUCTION**			
Rationale	3	Describe the rationale for the review in the context of what is already known.	4
Objectives	4	Provide an explicit statement of questions being addressed with reference to participants, interventions, comparisons, outcomes, and study design (PICOS).	4-5
**METHODS**			
Protocol and registration	5	Indicate if a review protocol exists, if and where it can be accessed (e.g., Web address), and, if available, provide registration information including registration number.	2
Eligibility criteria	6	Specify study characteristics (e.g., PICOS, length of follow-up) and report characteristics (e.g., years considered, language, publication status) used as criteria for eligibility, giving rationale.	6
Information sources	7	Describe all information sources (e.g., databases with dates of coverage, contact with study authors to identify additional studies) in the search and date last searched.	5
Search	8	Present full electronic search strategy for at least one database, including any limits used, such that it could be repeated.	5 & supp file 1
Study selection	9	State the process for selecting studies (i.e., screening, eligibility, included in systematic review, and, if applicable, included in the meta-analysis).	6
Data collection process	10	Describe method of data extraction from reports (e.g., piloted forms, independently, in duplicate) and any processes for obtaining and confirming data from investigators.	6
Data items	11	List and define all variables for which data were sought (e.g., PICOS, funding sources) and any assumptions and simplifications made.	6
Risk of bias in individual studies	12	Describe methods used for assessing risk of bias of individual studies (including specification of whether this was done at the study or outcome level), and how this information is to be used in any data synthesis.	6 & supp table 1
Summary measures	13	State the principal summary measures (e.g., risk ratio, difference in means).	7 - 8
Synthesis of results	14	Describe the methods of handling data and combining results of studies, if done, including measures of consistency (e.g., I^2) for each meta-analysis.	7 - 8

For peer review only

PRISMA 2009 Checklist

Section/topic	#	Checklist item	Reported on page #
Risk of bias across studies	15	Specify any assessment of risk of bias that may affect the cumulative evidence (e.g., publication bias, selective reporting within studies).	Sup Table 1
Additional analyses	16	Describe methods of additional analyses (e.g., sensitivity or subgroup analyses, meta-regression), if done, indicating which were pre-specified.	-
**RESULTS**			
Study selection	17	Give numbers of studies screened, assessed for eligibility, and included in the review, with reasons for exclusions at each stage, ideally with a flow diagram.	8, figure 1and Table 1
Study characteristics	18	For each study, present characteristics for which data were extracted (e.g., study size, PICOS, follow-up period) and provide the citations.	8 - 9
Risk of bias within studies	19	Present data on risk of bias of each study and, if available, any outcome level assessment (see item 12).	9 supp Table 1
Results of individual studies	20	For all outcomes considered (benefits or harms), present, for each study: (a) simple summary data for each intervention group (b) effect estimates and confidence intervals, ideally with a forest plot.	-
Synthesis of results	21	Present results of each meta-analysis done, including confidence intervals and measures of consistency.	8-13 Figures 2-9
Risk of bias across studies	22	Present results of any assessment of risk of bias across studies (see Item 15).	Supp Table 1
Additional analysis	23	Give results of additional analyses, if done (e.g., sensitivity or subgroup analyses, meta-regression [see Item 16]).	Table 10
**DISCUSSION**			
Summary of evidence	24	Summarize the main findings including the strength of evidence for each main outcome; consider their relevance to key groups (e.g., healthcare providers, users, and policy makers).	13-16
Limitations	25	Discuss limitations at study and outcome level (e.g., risk of bias), and at review-level (e.g., incomplete retrieval of identified research, reporting bias).	15-16
Conclusions	26	Provide a general interpretation of the results in the context of other evidence, and implications for future research.	13-15
**FUNDING**			
Funding	27	Describe sources of funding for the systematic review and other support (e.g., supply of data); role of funders for the systematic review.	17

For peer review only

PRISMA 2009 Checklist

From: Moher D, Liberati A, Tetzlaff J, Altman DG, The PRISMA Group (2009). Preferred Reporting Items for Systematic Reviews and Meta-Analyses: The PRISMA Statement. PLoS Med 6(6): e1000097. doi:10.1371/journal.pmed1000097

For more information, visit: **www.prisma-statement.org**.

For peer review only

For peer review only - http://bmjopen.bmj.com/site/about/guidelines.xhtml

BMJ Open

Treatments for gestational diabetes: A systematic review and meta-analysis

Journal:	*BMJ Open*
Manuscript ID	bmjopen-2016-015557.R1
Article Type:	Research
Date Submitted by the Author:	07-Mar-2017
Complete List of Authors:	farrar, diane; Bradford Institute for Health research, Maternal and Child health<br>Simmonds, Mark; University of York, Centre for Reviews and Dissemination<br>Bryant , Maria ; University of Leeds,<br>Sheldon, Trevor; University of York, health Sciences<br>tuffnell, derek; Bradford Royal Infirmary, Women's and Newborn unit<br>Golder, Su; University of York, Department of Health Sciences<br>Lawlor, Debbie; Department of Social Medicine, University of Bristol, MRC Integrative Epidemiology Unit
**Primary Subject Heading**:	Obstetrics and gynaecology
Secondary Subject Heading:	Diabetes and endocrinology
Keywords:	gestational diabetes, packages of care, insulin, metformin, glibenclamide, systematic review

SCHOLARONE™
Manuscripts

For peer review only

Treatments for gestational diabetes: A systematic review and meta-analysis

Diane Farrar*, Bradford Institute for Health Research, Bradford Royal Infirmary and Department of Health Sciences, University of York

Mark Simmonds, Centre for Reviews and Dissemination, University of York

Maria Bryant, Leeds Institute of Clinical Trials Research, University of Leeds

Trevor A Sheldon, Hull York Medical School, University of York

Derek Tuffnell, Bradford Women's and Newborn Unit, Bradford

Su Golder, Department of Health Sciences, University of York

Debbie A Lawlor, MRC Integrative Epidemiology Unit and School of Social and Community Medicine at the University of Bristol

*Corresponding author

Email: Diane.Farrar@bthft.nhs.uk

Tel: 01274 383416

Abstract word count: 371

Main text word count: 3723

Running title: Treatments for gestational diabetes

For peer review only - http://bmjopen.bmj.com/site/about/guidelines.xhtml

For peer review only

Abstract

Objective: To investigate the effectiveness of different treatments for GDM.

Design: Systematic review, meta-analysis and network meta-analysis

Methods: Data sources were searched up to July 2016 and included: MEDLINE and Embase. Randomised trials comparing treatments for GDM (packages of care (dietary and lifestyle interventions with pharmacological treatments as required), insulin, metformin, glibenclamide (glyburide)), were selected by two authors and double checked for accuracy. Outcomes included: large for gestational age; shoulder dystocia; neonatal hypoglycaemia; Caesarean section and pre-eclampsia. We pooled data using random-effects meta-analyses and used Bayesian network meta-analysis to compare pharmacological treatments (i.e. including treatments not directly compared within a trial).

Results: Forty two trials were included, the reporting of which was generally poor with unclear or high risk of bias. Packages of care varied in their composition and reduced the risk of most adverse perinatal outcomes compared to routine care (e.g. large for gestational age: RR 0.58 (95% CI 0.49-0.68; I^2 =0%; trials=8; participants =3462). Network meta-analyses suggest metformin had the highest probability of being the most effective treatment in reducing the risk of most outcomes compared to insulin or glibenclamide.

Conclusions: Evidence shows packages of care are effective in reducing the risk of most adverse perinatal outcomes. However trials often include few women, are poorly reported with unclear or high risk of bias and report few outcomes. The contribution of each treatment within the packages of care remains unclear. Large well-designed and conducted trials are urgently needed.

Systematic Review Registration: PROSPERO CRD42013004608

For peer review only - http://bmjopen.bmj.com/site/about/guidelines.xhtml

Key words: gestational diabetes; systematic review; meta-analysis; network analysis, treatments; packages of care; insulin; metformin; glibenclamide (glyburide)

Strengths and limitations of this study:

This systematic review evaluates available interventions for the treatment of gestational hyperglycaemia and includes a network meta-analysis comparing all pharmacological treatments for gestational diabetes.

A large number of trials conducted in varied populations have been included.

For some comparisons the numbers of trials included were few and outcomes reported were few.

Trial quality was generally poor with subsequent high or unclear risk of bias.

For peer review only

For peer review only - http://bmjopen.bmj.com/site/about/guidelines.xhtml

Introduction

Treatment of gestational diabetes (GDM) aims to reduce hyperglycaemia and in turn reduce the risk of adverse perinatal outcomes including: large for gestational age (LGA), macrosomia, shoulder dystocia, neonatal hypoglycaemia and the need for Caesarean section. Diet modification is often used as first-line treatment and if partly or wholly unsuccessful, or where women have substantially elevated glucose at diagnosis, pharmacological treatments (metformin, glibenclamide (glyburide) and/or insulin) are offered.

Previous systematic reviews have investigated the effectiveness of treatments for GDM,[1-15] Although results from these reviews generally indicate that treatment reduces the risk of adverse perinatal outcomes, the searches have variable inclusion criteria and were undertaken between 2009[1,5] and 2014[2-4,7,16 11 6,8,10,16] with three reviews with searches in 2015[9,14,15] and since then several trials have been published and recommended criteria for GDM diagnosis has changed. Some reviews have included observational studies and most do not review all treatments, with the exception of the Cochrane treatments review[1] (which is now out of date and has been divided for future updates) and the UK NICE guideline.[16] Consequently most previous reviews do not provide an assessment of all available treatments and most have not used a network meta-analysis to determine the most effective pharmacological treatment across all alternatives included in any randomised trial (RCT).

The aim of this study was to systematically review, and where appropriate pool all results from RCTs of the effect of any treatment on GDM and to determine which treatment is most effective.

Methods

We conducted a systematic review, meta-analysis and network meta-analysis to evaluate whether treatments for GDM reduce the risks of adverse perinatal outcomes and to compare the effectiveness of these treatments.

For peer review only - http://bmjopen.bmj.com/site/about/guidelines.xhtml

This review and meta-analysis was conducted in accordance with Cochrane systematic reviews[17] and the Centre for Reviews and Dissemination recommendations,[18] we have reported our findings following the PRISMA reporting guidelines (see research checklist).[19] This review forms part of a larger Health Technology Assessment report of the diagnosis and management of GDM.[20]

Patient involvement

The outcomes we included were from the Cochrane Pregnancy and Childbirth Group's standardised outcomes for reviews of diabetes in pregnancy. Women who had experienced or had the potential to experience GDM contribute to the design and appraisal of this group's methods and reviews and therefore have influenced the design of this review and outcomes examined.[21]

Search methods

The search strategies were designed to identify records of RCTs of treatment for women with GDM, added to search sources since the search date (July 2011, trials awaiting classification) of the Cochrane 'treatments for GDM' review.[1] The bibliographic databases searched were MEDLINE and MEDLINE in Process, Embase and the Cochrane Central Register of Controlled Trials. Strategies were not restricted by language and were developed using a combination of subject indexing terms and free text search terms in the title and abstract fields. Searches were first conducted in September 2013 and updated in October 2014 and 6th July 2016, using the same search strategies. Information on studies in progress was sought by searching relevant trial registers including ClinicalTrials.gov.

We also searched previously published systematic reviews to ensure any eligible RCTs from these were included in our review if eligible.[2-9] In addition we checked the references of included journal articles. An example of search terms for MEDLINE are included in Supplementary file 1.

For peer review only - http://bmjopen.bmj.com/site/about/guidelines.xhtml

Study selection: Inclusion and exclusion criteria

We included RCTs in which women with diagnosed GDM or impaired glucose tolerance (IGT) (using any definition) were randomised to a treatment designed to lower blood glucose (pharmacological or dietary modification) compared to routine antenatal care (however defined by the trial) or another treatment. Trials including women with pre-existing diabetes were excluded. Trials had to report effects on adverse perinatal outcomes. Included outcomes (defined in any way by the trials) were: gestational age at birth; birth weight (BW); macrosomia; large for gestational age (LGA); shoulder dystocia; preterm birth (less than 37 weeks gestation); neonatal hypoglycaemia; admission to neonatal intensive care unit (NICU); Caesarean section (elective or emergency); pre-eclampsia; pregnancy-induced hypertension (PIH); induction of labour; instrumental birth (forceps or ventouse); Apgar score at five minutes; and negative treatment effects (e.g. gastrointestinal upset, wellbeing). Data on side effects and quality of life measures were also examined. Conference abstracts and letters to journals were eligible for inclusion if they reported sufficient outcome data.

Data extraction and risk of bias assessment

Title and abstract screening and then full text screening was performed by two reviewers (DF, MS, MB or SG) with disagreements resolved by consensus or by the third reviewer. The risk of bias of the included trials was assessed using the Cochrane risk of bias tool,[22] which considers: sequence generation, allocation concealment, blinding of participants and medical staff to treatment allocation, blinding of assessors, loss to follow up, selective reporting of outcomes and other sources of bias. Each criterion was classified as being at low or high risk of bias, or unclear. Two reviewers independently assessed all criteria (DF, MS or SG).

For peer review only - http://bmjopen.bmj.com/site/about/guidelines.xhtml

Statistical analysis

Trials were divided into categories according to the included treatments: (1) insulin versus metformin; (2) insulin versus glibenclamide (glyburide); (3) metformin versus glibenclamide; (4) packages of care: diet or dietary advice with or without exercise or glucose monitoring, with or without supplemental metformin, glibenclamide or insulin, compared to routine antenatal care; (5) comparisons of different dietary modifications.

For dichotomous outcomes, the relative risk comparing each group, with its 95% confidence interval, was calculated from the numbers of outcome events in each randomised group and the number randomised to each group. For continuous outcomes, the difference in means between groups was calculated from the mean and standard deviation of the outcome. For each outcome, and within each of the treatment categories, relative risks or differences in means were pooled in random-effects DerSimonian-Laird meta-analyses.[23] Heterogeneity was assessed using I^2.[24] Analyses were performed to investigate differences in risk of outcomes across varying degrees of hyperglycaemia (defined by a positive/negative GDM screening and diagnostic test). Because of the large number of treatments and outcome comparisons, pooled estimates only are presented in the main paper. Tests for publication bias were considered, but not performed, because there were insufficient trials in any meta-analysis for such tests to be reliable.

We also conducted a network meta-analysis to combine information across multiple treatments simultaneously, this combines direct and indirect data to improve the estimation of the effectiveness of treatments and specifically to try to estimate which is the most effective of a number of different treatment options.[25-28] Analyses were undertaken for each dichotomous outcome using a Bayesian approach, based on the models originally created by Lu and Ades,[29] using the OpenBUGS[30] software. The model has a “Binominal-normal” structure; that is, events were assumed to follow a binomial distribution, with log odds and random effects being normally

For peer review only - http://bmjopen.bmj.com/site/about/guidelines.xhtml

distributed. Vague normal priors (mean 0, variance 10000) were used except for heterogeneity, where an inverse-gamma (0.1, 0.1) distribution was used. The model fit and consistency were assessed by comparing the results to the meta-analyses comparing each treatment directly.

Each model generated a comparison between treatments, expressed as an odds ratio, and as a percentage indicating the probability that the treatment was the best treatment to reduce the incidence of the adverse outcome. Odds ratios were used to ensure model stability, because log odds ratios more closely follow a normal distribution than relative risks. The probabilities of being most effective treatment were calculated from the posterior odds as part of the Bayesian model developed by Lu and Ades.[29] This approach was not possible for continuously measured outcomes and so was not undertaken for gestational age, birthweight and Apgar score. As there were no trials comparing diet modification to pharmacological treatments, diet modification could not be included in the network meta-analyses.

Results

Details of included and excluded trials

12234 citations were identified by the original and the two update searches. These citations were combined with three additional citations identified by previous systematic reviews conducted prior to our first searches .[1-5] Following de-duplication and inclusion of additional records, 6437 citations were reviewed. Of these, 214 were judged potentially eligible based on title and abstract. After obtaining the full text publications and assessing eligibility, 42 trials were included and 35 of these were combined in at least one meta-analysis (Figure 1).

Having extracted data from the RCTs assessing packages of care and dietary intervention comparisons (Table 1), we decided that it was not appropriate to pool results from trials comparing

For peer review only - http://bmjopen.bmj.com/site/about/guidelines.xhtml

dissimilar dietary modification interventions (Table 1). Packages of care included various combinations of interventions, however all packages of care compared with routine care trial results were pooled in meta-analyses.

We included eight publications not included in any previous published review. One compared metformin and insulin,[31] one, glibenclamide and insulin,[32] four, packages of care with routine care[33-36] and two compared different dietary modification interventions.[37,38] Six of these trials were reported after the search dates of the previous reviews and were published in 2014 or 2015, the remaining two trials (dietary modification interventions or packages of care) did not fulfil other review's inclusion criteria. Few trials reported side effects or measures of participant satisfaction or wellbeing.

Trials generally included women with GDM diagnosed following a 75g or 100g oral glucose tolerance test (OGTT) using a variety of international [39,40,41] and locally [42,43] recommended thresholds, though some included women with 'mild or borderline' GDM (positive oral glucose tolerance test (OGCT), negative OGTT) and others included women with 'impaired glucose tolerance' (IGT), current diagnostic criteria[16,44] however may now consider these women as having GDM rather than a separate and milder condition.

Quality –Risk of bias assessment

Overall, reporting of, and many aspects of trial quality, was poor with the result that risk of bias was generally unclear or high (Supplementary Table 1). The randomisation procedure and group allocation was rarely described, although all trials reported that participants were 'randomised'. Blinding of participants, medical staff and outcome assessors was generally not reported, but as most trials include some additional intervention above routine care such as diet advice or a pharmacological treatment, it is probable that participants and most clinicians could not be blinded,

For peer review only - http://bmjopen.bmj.com/site/about/guidelines.xhtml

For peer review only

though outcome assessment could have been. Most trials had reasonably complete outcome data and loss to follow-up was low, though for some trials analysis was not conducted on an intention to treat basis (so the analysis did not include all women randomised). Selective reporting was assessed as minimal as the majority of trials presented results for all pre-specified outcomes (the possibility that some trials collected data on outcomes, but did not report them cannot be ruled out however).

Generally, women were eligible for inclusion in trials evaluating pharmacological treatments if they were unable to achieve adequate glycaemic control with dietary and lifestyle management. Therefore there is the possibility that those included may have had more severe or refractory hyperglycaemia or may adhere less well to lifestyle interventions than those women who did not require pharmacological treatments to control hyperglycaemia. The specific criteria for the addition of supplemental insulin in trials were often not reported, though some trials did report that supplemental insulin was prescribed if 'glycaemic control was not achieved by participants'. It is probable that thresholds for what is defined as 'good' control differed between trial centres (if multi-site) and trials.

Packages of care and dietary modification trials

Twelve trials evaluated a package of care (a combination of treatments starting with dietary modification and/or exercise and/or monitoring and/or supplemental pharmacological treatments) (Table 1)[33-36,45-52] compared to routine care. Data from these 12 trials are combined in at least one meta-analysis (Figure 2a, 2b).

Seven trials[37,38,53-57] evaluated a variety of dietary modifications and compared them to other dietary modifications (Table 1). The composition of each dietary modification was generally well reported, however the interventions and comparisons were too diverse to allow pooling of data. There was no

For peer review only - http://bmjopen.bmj.com/site/about/guidelines.xhtml

evidence that one type of dietary modification was superior over another, though trials included few women (Supplementary Figure 1, 2). None of these seven trials reported side effects or quality of life measures.

The composition of the dietary modification was poorly reported in the 'packages of care' trials (the 12 trials included in the meta-analyses). Overall (in all packages of care and dietary modification trials), 10 out of 19 trials reported that insulin was provided if required, in one trial insulin was only provided if needed in the intervention group and for the remainder it was unclear or not reported if supplemental insulin was provided. The screening and diagnostic tests, criteria and glucose thresholds used to define GDM (and included/exclude women in the trials) varied across the trials (Table 1). For the meta-analysis the varying forms of dietary modification and/or pharmacological treatment use was not examined.

Packages of care (starting with dietary modification and possibly including monitoring and pharmacological interventions) reduced the risk of shoulder dystocia by 60%, LGA and macrosomia by around 50%, pre-eclampsia by 20% and the incidence of Caesarean section by 10% compared to routine care (Figure 2a) though for pre-eclampsia and Caesarean section the confidence intervals included the null value. BW was reduced by approximately 110g in the packages of care compared to routine care group (Figure 2b). The degree of heterogeneity (I^2) varied by outcome from 0% to 77%. No 'packages of care trial' reported side effects; two trials reported quality of life scores[47,48] indicating higher (better) quality of life scores for women in the intervention compared to the routine care group.

Trials comparing metformin with insulin

Eleven trials compared metformin with Insulin (Table 2).[31,43,58-66] However most trials reported supplemental insulin use in the metformin group with the exception of two trials.[31,64] The risk of

For peer review only - http://bmjopen.bmj.com/site/about/guidelines.xhtml

most outcomes, including LGA, macrosomia, NICU admission, neonatal hypoglycaemia, pre-eclampsia, PIH and induction of labour (IOL), was lower in those randomised to metformin rather than insulin; instrumental delivery was greater in those randomised to insulin (Figure 2c). Birthweight, gestational age and Apgar score as continuous measurements did not differ notably between the two treatments (Figure 2d). Six trials reported the proportion of women with metformin associated gastrointestinal upset (between 4% to 46%).[58-60,63,65,66] No trial reported quality of life measures.

Trials comparing glibenclamide (glyburide) with insulin

Nine trials compared glibenclamide with insulin (Table 3).[32,67-74] Figure 2e shows the relative risks of dichotomous outcomes, suggesting insulin may be relatively more effective than glibenclamide in reducing the risk of several adverse outcomes, confidence intervals are wide and include the null value however. There was no difference between insulin and glibenclamide for continuous outcomes (Figure 2f). One trial reported that glibenclamide was associated with side effects in 3/48 (6%) of women.[72] No trial reported quality of life measures.

Trials comparing glibenclamide (glyburide) with metformin

Only three trials were identified that directly compared glibenclamide with metformin and these were relatively small trials including between 149 and 200 women (Table 4).[75-77] Figure 2g shows the risk of dichotomous and Figure 2h continuous outcomes. These suggest metformin is more effective at reducing risk of LGA and possibly macrosomia. However, for several of the outcomes (for example LGA) only data from one of these trials is available, it is therefore not possible to make robust conclusions about the relative benefits of metformin and glibenclamide from these direct comparisons. No trials reported side effects or quality of life measures.

For peer review only - http://bmjopen.bmj.com/site/about/guidelines.xhtml

Network meta-analysis comparing glibenclamide (glyburide), insulin and metformin

Figure 3 shows the relationship of treatment comparisons and Table 5 shows the estimated probability of a treatment being the most effective at reducing the risk of each dichotomous outcome. Only dichotomous outcomes reported in at least two glibenclamide trials (either in comparison to insulin or metformin) were included in these analyses to ensure there were sufficient trials (and participants) included. When all three treatments are jointly compared, these analyses suggest that, for all outcomes, with the exception of Caesarean section, metformin is most likely to be the most effective treatment, with its probability of being most effective in reducing risk being 96.3, 94.0%, 92.8%, 84.0% and 61.2% respectively for neonatal hypoglycaemia, macrosomia, LGA, pre-eclampsia and admission to NICU (the probability of being most effective for reducing risk of Caesarean section was 9.7% for metformin, glibenclamide was most likely to be most effective at reducing the risk of Caesarean section (79.9%)). The results of the network meta-analysis (Figure 4) are consistent with the direct comparisons between treatments shown in Figures 2a to 2h, suggesting that metformin is more effective than insulin or gliblenclamide at reducing the majority of adverse outcomes. However, many of these comparisons are based on small numbers and have wide confidence intervals that sometimes include the null value.

Discussion

The key finding of our review is that, despite understanding of hyperglycaemia/GDM and its relationship to adverse perinatal outcomes having existed for at least seven decades,[78] and 42 RCTs completed on its treatment; trials are still being conducted that are of limited size and of poor quality (with subsequent unclear or high risk of bias) and therefore which treatment is most effective remains unclear. Given the changing characteristics of the population and the lower fasting diagnostic threshold (compared to previous criteria[40]) recommended by the IADPSG[44] and UK NICE,[16] it is important to understand how treatments affect outcomes for these women. Trials do not always report GDM diagnostic criteria clearly and this is important considering the potential influence on

For peer review only - http://bmjopen.bmj.com/site/about/guidelines.xhtml

GDM population size and the magnitude of effect.[16,44] Our detailed review, including only evidence from RCTs, provides some support for a ‘step up approach’ in the treatment of hyperglycaemia, from dietary interventions, through addition of metformin (in preference to glibenclamide (glyburide)) through to addition of insulin. Considering that hyperglycaemia in pregnancy has various causes and many women will be treated successfully with diet and lifestyle interventions (because lower thresholds lead to less severe hyperglycaemia being classified as GDM) using an integrated individual approach to its management, is likely to work best, though trials and reviews continue to be conducted that pay little attention to the influence of non-pharmacological treatments for GDM and often do not provide information on the severity of hyperglycaemia in treatment groups.

We have taken a pragmatic approach to evaluating the many trials examining treatment packages of care for women diagnosed with hyperglycaemia/GDM so that our results will be generalisable to most clinical situations. Several previous reviews have focused exclusively on pharmacological treatments,[2,6,8,9,12-15] however others have also suggested packages of care with a ‘step up’ approach are most effective.[1,3-5] The severity of hyperglycaemia may influence the effectiveness of a treatment, however many trials do not report treatment subgroup baseline glycaemic levels (for example diet only, diet and metformin or insulin, or metformin with supplementary insulin).[34-36,45,47,48,51,62-65,79] For those trials reporting baseline glycaemic levels by treatment subgroup there is inconsistency, with some reporting significant differences between groups [59,66] and others reporting no difference.[43,58,60] Understanding of treatment effects would be improved if baseline OGTT levels were presented by treatment subgroup in future trials.

The number of trials and women included in previous reviews varies. One recent review had broadly similar inclusion criteria to ours, comparing any package of care for the treatment of GDM with no treatment (routine care) and included five trials with 2643 women.[3] Our review includes all these trials, plus a further seven (included in the meta-analysis) increasing the number of women to 4512 and indicating that RCTs in this area continue to be conducted, but not with the size or quality that

For peer review only - http://bmjopen.bmj.com/site/about/guidelines.xhtml

allows us to have a robust evidence base for the treatment of GDM in a contemporary population. Pooled estimates are generally consistent across reviews of packages of care irrespective of the number of trials included, because estimates are driven in all reviews by the two largest, which are also the highest quality trials, however these trials were conducted in populations using diagnostic criteria that would provide populations with more severe hyperglycaemia (and therefore the potential for a larger effect size).[47,50] For example, our analysis shows the risk of macrosomia is halved when a package of care is provided compared to routine care (11 trials, RR 0.49, 95% CI 0.39-0.62), confirming estimates from the most recent previous review (RR 0.50 95% CI 0.35-0.71).[3] These two large and well-conducted RCTs were published in 2005 and 2009,[47,50] and since then several smaller and poorer quality trials have been published. These two previous large well-conducted trials cannot provide precise estimates of effect on the wider range of adverse outcomes and for women diagnosed using more recently recommended criteria. Hence, we feel it is important to place a moratorium on further small RCTs in this area and that funders should consider commissioning a multi-centre large-scale RCT with adequate power to determine the effect and cost-effectiveness of different packages of care on adverse outcomes in women with GDM.

The evidence to support metformin use, though encouraging has certain weaknesses. Firstly although there is a general 'trend' in favour of metformin use over insulin and glibenclamide (glyburide), confidence intervals are wide, in both the direct and network meta-analysis comparing each two-way treatment effect. Secondly the reporting of trial methods was generally poor with 'unclear or high risk of bias' and many trials included relatively few women and reported few outcomes. Thirdly, in most trials directly comparing metformin with insulin, women receiving metformin were also given supplemental insulin 'if required'; in one of the largest trials this equated to 46% of the metformin group.[59] Therefore our results more appropriately relate to metformin's greater effectiveness as a first-line treatment for GDM rather than a standalone treatment compared to insulin.

For peer review only - http://bmjopen.bmj.com/site/about/guidelines.xhtml

In addition to being an effective first-line pharmacological treatment for GDM, metformin may also be preferred by women as it is administered orally and can be stored at room temperature, compared to insulin that requires subcutaneous injection and refrigerated storage. Metformin is sometimes associated with gastrointestinal upset, which may affect compliance and quality of life.

Few trials have reported side effects or measures of participant satisfaction or wellbeing, all important outcomes that have the potential to impact health and therefore should be evaluated. Recent guidance[16,44] recommends lower glucose thresholds compared to those previously recommended to diagnose GDM[39,40] (and used in the included trials). Therefore it is possible that a greater proportion of women diagnosed with GDM will require only diet modification or less 'intensive' management compared to those previously diagnosed with GDM, because their hyperglycaemia is less severe. There is a continuum of increasing risk of adverse outcomes across the spectrum of glucose however[80,81] therefore interventions to reduce hyperglycaemia even at lower glucose levels are likely to improve outcomes, but this needs confirming by large well-designed RCTs.

Strengths and limitations

This systematic review and meta-analysis includes a large number of trials with varied populations, and examines the effectiveness of treatment packages and diets as well as individual pharmacological treatments for reducing the risk of adverse perinatal outcomes.

For some comparisons, trials and numbers of women were few, as were outcomes reported. Trial quality was generally poor with subsequent high or unclear risk of bias. GDM diagnostic criteria varied across trials and recently recommended thresholds are lower now compared to when most included trials were conducted.

For peer review only - http://bmjopen.bmj.com/site/about/guidelines.xhtml

Lower glucose threshold criteria recommended by the International Association of Diabetes and Pregnancy Study Groups[44] and subsequently endorsed by the World Health Organization[82] aim to identify offspring at risk of obesity through its association with LGA (birth weight >90th percentile), cord C-peptide >90th percentile and percentage body fat >90th percentile. However there are no trials that have used these criteria and the classification of less severe hyperglycaemia when lower glucose thresholds are used to diagnose GDM may reduce the magnitude of the effect of interventions, compared to those reported by earlier trials using higher glucose thresholds. There has also been no longer-term follow up conducted to evaluate the treatment of GDM and the effects on risk of offspring. Importantly, few of the trials that we reviewed had reported side effects or measures of participant satisfaction or wellbeing.

Implications for practice

This review provides reassurance that a package of care where a 'step up' approach of firstly providing dietary and lifestyle advice, then adding supplementary metformin or insulin if glucose levels are not adequately controlled, is a reasonable and effective approach compared to providing just routine antenatal care, particularly with regards to reducing the risk of LGA. However, it has also highlighted the general poor quality of recent small RCTs that do not improve the evidence base, but subject women with GDM to unnecessary 'experimentation' and are a cost to society.

Metformin seems to be an effective alternative to insulin, if diet modification inadequately controls hyperglycaemia, however supplemental insulin may be required in up to 50% of women.[59] There is a need to cease further small RCTs in this area and conduct large well-designed RCTs that clarify the most effective treatment across a range of outcomes, including those that are likely to be important to women such as quality of life measurements and those identified by the Cochrane Pregnancy and Childbirth Group (CPCG) as being essential for trials and reviews of diabetes in pregnancy. These

For peer review only - http://bmjopen.bmj.com/site/about/guidelines.xhtml

should be incorporated into current diagnostic criteria and ideally look at longer-term outcomes in mothers and offspring.

Acknowledgements

Thank you to Julie Glanville and Mick Arber of the York Health Economics Consortium, University of York, UK, who carried out the searches.

Details of ethics approval

This study did not require ethical approval as the data used have been published previously, and hence are already in the public domain.

Contribution to authorship

DF, MS, DAL and TAS designed the study. MS wrote the statistical analysis plan. DF monitored the review process. DF, MS, MB, DAL, TAS, DT and FD interpreted the data, DF, MS, MB and SG assessed studies for inclusion. MS cleaned and analysed the data, DF wrote the draft paper. All authors have approved the final version. Diane Farrar is guarantor and takes responsibility for the content of this article.

Funding

This work was supported by the National Institute for Health Research (NIHR), Health Technology Assessment (HTA) programme, project number 11/99/02. DF holds a NIHR Post-doctoral Research Fellowship award (PD-2014-07-019). DAL works in a Unit that is supported by the University of Bristol and UK Medical Research Council ((MC_UU_12013/5) and she holds a NIHR Senior Investigator award (NF-SI-0611-10196).The views and opinions expressed therein are those of the authors and do not necessarily reflect those of the HTA, NIHR, MRC, UK National Health Service (NHS) or the Department of Health.

For peer review only - http://bmjopen.bmj.com/site/about/guidelines.xhtml

Data sharing

Extracted data are available upon request to the corresponding author

Competing interests: All authors have completed the ICMJE uniform disclosure form at www.icmje.org/coi_disclosure.pdf and declare: no support from any organisation for the submitted work; no financial relationships with any organisations that might have an interest in the submitted work in the previous three years; no other relationships or activities that could appear to have influenced the submitted work.

For peer review only

For peer review only - http://bmjopen.bmj.com/site/about/guidelines.xhtml

References

1. Alwan N, Tuffnell D, West J. Treatments for gestational diabetes. *Cochrane Database of Syst Rev*. 2009; Issue 3:CD003395.

2. Gui J, Liu Q, Feng L. Metformin vs insulin in the management of gestational diabetes: a meta-analysis. *PloS one*. 2013; 8(5): e64585.

3. Hartling L, Dryden DM, Guthrie A, Muise M, Vandermeer B, Donovan L. Benefits and harms of treating gestational diabetes mellitus: a systematic review and meta-analysis for the U.S. Preventive Services Task Force and the National Institutes of Health Office of Medical Applications of Research. *Ann Intern Med*. 2013; 159(2): 123-9.

4. Falavigna M, Prestes I, Schmidt MI, Duncan BB, Colagiuri S, Roglic G. Impact of gestational diabetes mellitus screening strategies on perinatal outcomes: a simulation study. *Diabetes Res Clin Pract*. 2012; 99(3): 358-65.

5. Horvath K, Koch K, Jeitler K, Matyas E, Ralf Bender R, Hilda Bastian H, et al. Effects of treatment in women with gestational diabetes mellitus: systematic review and meta-analysis. *BMJ*. 2010; 340: 1395-413.

6. Balsells M, García-Patterson A, Solà I, Roqué M, Gich I, Corcoy R. Glibenclamide, metformin, and insulin for the treatment of gestational diabetes: a systematic review and meta-analysis. BMJ [Journal Article] 2015 2015-01-21 16:06:23 [cited 350; Available from: http://www.bmj.com/bmj/350/bmj.h102.full.pdf

7. Han S, Crowther CA, Middleton P, Heatley E. Different types of dietary advice for women with gestational diabetes mellitus. *Cochrane Database Syst Rev*. 2013; (3).

8. Jiang Y-F, Chen X-Y, Ding T, Wang X-F, Zhu Z-N, Su S-W. Comparative efficacy and safety of OADs in management of GDM: network meta-analysis of randomized controlled trials. *J Clin Endocrinol Meteb*. 2015; 100(5): 2071-80.

9. Amin M, Suksomboon N, Poolsup N, Malik O. Comparison of glyburide with metformin in treating gestational diabetes mellitus: a systematic review and meta-analysis. *Clin Drug Investig*. 2015; 35(6): 343-51.

10. Kitwitee P, Limwattananon S, Limwattananon C, Waleekachonlert O, Ratanachotpanich T, Phimphilai M, et al. Metformin for the treatment of gestational diabetes: An updated meta-analysis. *Diabetes Res Clin Pract*. 2015; 109(3): 521-32.

11. Zhao L-P, Sheng X-Y, Zhou S, Yang T, Ma L-Y, Zhou Y, et al. Metformin versus insulin for gestational diabetes mellitus: a meta-analysis. *Br J Clin Pharmacol*. 2015; 80(5): 1224-34.

12. Su DF, Wang XY. Metformin vs insulin in the management of gestational diabetes: A systematic review and meta-analysis. *Diabetes Res Clin Pract*. 2014; 104(3): 353-7.

13. Zhu B, Zhang L, Fan YY, Wang L, Li XG, Liu T, et al. Metformin versus insulin in gestational diabetes mellitus: a meta-analysis of randomized clinical trials. *Ir J Med Sci*. 2016; 185(2): 371-81.

14. Feng Y, Yang H. Metformin – a potentially effective drug for gestational diabetes mellitus: a systematic review and meta-analysis. *J Matern Fetal Neonatal Med*. 2016; http://dx.doi.org/10.1080/14767058.2016.1228061: 1-8.

15. Butalia S, Gutierrez L, Lodha A, Aitken E, Zakariasen A, Donovan L. Short- and long-term outcomes of metformin compared with insulin alone in pregnancy: a systematic review and meta-analysis. *Diabetic Med*. 2017; 34(1): 27-36.

16. National Institute for Health and Care Excellence. Diabetes in pregnancy: management of diabetes and its complications from preconception to the postnatal period. *National collaborating centre for Women's and Children's Health*. 2015; https://www.nice.org.uk/guidance/ng3.

17. Higgins JPT, Green S, editors. Cochrane Handbook for Systematic Reviews of Interventions Version 5.1.0 [updated March 2011]. *The Cochrane Collaboration*. 2011; Available from www.cochrane-handbook.org.

18. Centre for Reviews and Dissemination. Systematic reviews: CRD's guidance for undertaking a systematic review York: CRD, University of York

For peer review only - http://bmjopen.bmj.com/site/about/guidelines.xhtml

2009.
19. Preferred Reporting Items for Systematic Reviews and Meta-Analyses. PRISMA. *http://wwwprisma-statementorg/*. http://www.prisma-statement.org/.
20. Farrar D, Simmonds M, Griffin S, Duarte A, Lawlor DA, Sculpher M, et al. The identification and treatment of women with hyperglycaemia in pregnancy: an analysis of individual participant data, systematic reviews and an economic evaluation. *HTA*. 2013; 20(86).
21. Sakala C, Gyte G, Henderson S, Neilson JP, Horey D. Consumer-Professional Partnership to Improve Research: The Experience of the Cochrane Collaboration's Pregnancy and Childbirth Group. *Birth*. 2001; 28(2): 133-7.
22. The Cochrane Collaboration. The Cochrane Collaboration’s tool for assessing risk of bias. *http://ohgcochraneorg/sites/ohgcochraneorg/files/uploads/Risk%20of%20bias%20assessment%20toolpdf*. last accessed Jan 2014.
23. DerSimonian R, Laird N. Meta-analysis in clinical trials. *Control Clin Trials*. 1986; 7(3): 177-88.
24. Higgins JPT, Thompson SG. Quantifying heterogeneity in a meta-analysis. *Stats Med*. 2002; 21(11): 1539-58.
25. Bafeta A, Trinquart L, Seror R, Ravaud P. Reporting of results from network meta-analyses: methodological systematic review. *BMJ*. 2014; 348.
26. Lumley T. Network meta-analysis for indirect treatment comparisons. *Stat Med*. 2002; 21(16): 2313-24.
27. Song F, Altman DG, Glenny A-M, Deeks JJ. Validity of indirect comparison for estimating efficacy of competing interventions: empirical evidence from published meta-analyses. *BMJ*. 2003; 326(7387): 472.
28. Mills EJ, Thorlund K, Ioannidis JP. Demystifying trial networks and network meta-analysis. *BMJ*. 2013; 346(f2914).
29. Lu G, Ades AE. Combination of direct and indirect evidence in mixed treatment comparisons. *Statis Med*. 2004; 23(20): 3105-24.
30. openbugs. http://www.openbugs.net/w/FrontPage. last accessed January 2015.
31. Zinnat ANS, Shareen S, Rahman S. Can metformin be used in place of insulin for the treatment of GDM for low resource countries? *BJOG*. 2013; 120: 35.
32. Mirzamoradi M, Bakhtiyari M, Kimiaee P, Hosseini-Najarkolaei A, Mansournia MA. Investigating the effects of treatment based on single high blood glucose in gestational diabetes screening on maternal and neonatal complications. Arch Gynecol Obst; 2015. p. 687-95.
33. Deveer R, Deveer M, Akbaba E, Engin-Ustun Y, Aydogan P, Celikkaya H, et al. The effect of diet on pregnancy outcomes among pregnant with abnormal glucose challenge test. *Eur Rev Med Pharmacol Sci*. 2013; 17(9): 1258-61.
34. Fadl HE, Gardefors S, Hjertberg R, Nord E, Persson B, Schwarcz E, et al. Randomized controlled study in pregnancy on treatment of marked hyperglycemia that is short of overt diabetes. *Acta Obstet Gynecol Scand*. 2015; 94(11): 1181-7.
35. Yang X, Hsu-Hage B, Dong L, Shao P, Wang H, Tian H, et al. Intensive Diabetes Management May Improve Pregnancy. *Diabetes Care*. 2003; 26(1): 254.
36. Yang X, Tian H, Zhang F, Zhang C, Li Y, Leng J, et al. A randomised translational trial of lifestyle intervention using a 3-tier shared care approach on pregnancy outcomes in Chinese women with gestational diabetes mellitus but without diabetes. *J Transl Med*. 2014; (1).
37. Ma W-J, Huang Z-H, Huang B-X, Qi B-H, Zhang Y-J, Xiao B-X, et al. Intensive low-glycaemic-load dietary intervention for the management of glycaemia and serum lipids among women with gestational diabetes: a randomized control trial. *Public Health Nutr*. 2015; 18(8): 1506-13.
38. Yao J, Cong L, Zhu B, Wang T. Effect of dietary approaches to stop hypertension diet plan on pregnancy outcome patients with gestational diabetes mellitus. *Bangladesh J Pharmacol*. 2015; 10(4): 732-8.
39. American College of Obstetricians and Gynecologists. Practice Bulletin Clinical Management Guidelines for Obstetricians - Gynecologists. *Obstet Gynecol*. 2013; 122(2): 406- 16.

For peer review only - http://bmjopen.bmj.com/site/about/guidelines.xhtml

40. World Health Organization. Definition, diagnosis and classification of diabetes mellitus and its complications. Report of a WHO consultation. Part 1: diagnosis and classification of diabetes mellitus. 1999; http://apps.who.int/iris/bitstream/10665/66040/1/WHO_NCD_NCS_99.2.pdf.
41. American Diabetes Association. Standards of Medical Care in Diabetes - 2014. *Diabetes Care* 2014; 37(Supp 1): S14-80.
42. Ijäs H, Vääräsmäki M, Morin-Papunen L, Keravuo R, Ebeling T, Saarela T, et al. Metformin should be considered in the treatment of gestational diabetes: a prospective randomised study. *BJOG*. 2011; 118(7): 880-5.
43. Tertti K, Ekblad U, Koskinen P, Vahlberg T, Ronnemaa T. Metformin vs. insulin in gestational diabetes. A randomized study characterizing metformin patients needing additional insulin. *Diabetes Obes Metab*. 2013; 15(3): 246-51.
44. International Association of Diabetes and Pregnancy Study Groups Consensus panel. International Association of Diabetes and Pregnancy Study Groups Recommendations on the Diagnosis and Classification of Hyperglycemia in Pregnancy. *Diabetes Care*. 2010; 33(7): 676-82.
45. Bevier WC, Fischer R, Jovanovic L. Treatment of women with an abnormal glucose challenge test (but a normal oral glucose tolerance test) decreases the prevalence of macrosomia. Am J Perinatol; 1999. p. 269-75.
46. Bonomo M, Corica D, Mion E, Goncalves D, Motta G, Merati R, et al. Evaluating the therapeutic approach in pregnancies complicated by borderline glucose intolerance: a randomized clinical trial. *Diabet Med*. 2005; 22(11): 1536-41.
47. Crowther CA, Hiller JE, Moss JR, McPhee AJ, Jeffries WS, Robinson JS. Effect of treatment of gestational diabetes mellitus on pregnancy outcomes. *N Engl J Med*. 2005; 352(24): 2477-86.
48. Elnour AA, Mugammar IT, Jaber T, Revel T, McElnay JC. Pharmaceutical care of patients with gestational diabetes mellitus. J evaluat clin prac; 2008. p. 131-40.
49. Garner P, Okun N, Keely E, Wells G, Perkins S, Sylvain J, et al. A randomized controlled trial of strict glycemic control and tertiary level obstetric care versus routine obstetric care in the management of gestational diabetes: A pilot study. *Am J Obstet Gynecol*. 1997; 177(1): 190-5.
50. Landon MB, Spong CY, Thom E, Carpenter MW, Ramin SM, Casey B, et al. A multicenter, randomized trial of treatment for mild gestational diabetes. *N Engl J Med*. 2009; 361: 1339-48.
51. Li DF, Wong VC, O'Hoy KM, Yeung CY, Ma HK. Is treatment needed for mild impairment of glucose tolerance in pregnancy? A randomized controlled trial. BJOG; 1987. p. 851-4.
52. O'Sullivan JB, Gellis SS, Dandrow RV, Tenney BO. The Potential Diabetic and Her Treatment in Pregnancy. *Obstet Gynecol*. 1966; 27(5): 683-9.
53. Asemi Z, Samimi M, Tabassi Z, Esmaillzadeh A. The effect of DASH diet on pregnancy outcomes in gestational diabetes: a randomized controlled clinical trial. *Eur J Clin Nutr*. 2014; 68(4): 490-5.
54. Cypryk K, Kaminska P, Kosinski M, Petynska-Marczewska M, Lewinski A. A comparison of the effectiveness, tolerability and safety of high and low carbohydrate diets in women with gestational diabetes. Endokryno Pol; 2007. p. 314-9.
55. Louie JCY, Markovic TP, Perera N, Foote D, Petocz P, Ross GP, et al. A randomized controlled trial investigating the effects of a low-glycemic index diet on pregnancy outcomes in gestational diabetes mellitus. *Diabetes Care*. 2011; 34(11): 2341-6.
56. Moreno-Castilla C, Hernandez M, Bergua M, Alvarez MC, Arce MA, Rodriguez K, et al. Low-Carbohydrate Diet for the Treatment of Gestational Diabetes Mellitus: A randomized controlled trial. *Diabetes Care*. 2013; 36(8): 2233-8.
57. Rae A, Bond D, Evans S, North F, Roberman B, Walters B. A randomised controlled trial of dietary energy restriction in the management of obese women with gestational diabetes. Aust N Z J Obstet Gynaecol; 2000. p. 416-22.
58. Spaulonci CP, Bernardes LS, Trindade TC, Zugaib M, Francisco RPV. Randomized trial of metformin vs insulin in the management of gestational diabetes. *Am J Obstet Gynecol*. 2013; 209(1): 34e1-e7.

For peer review only - http://bmjopen.bmj.com/site/about/guidelines.xhtml

59. Rowan JA, Hague WM, Gao W, Battin MR, Moore MP. Metformin versus Insulin for the Treatment of Gestational Diabetes. *N Engl J Med*. 2008; 358(19): 2003-15.
60. Niromanesh S, Alavi A, Sharbaf FR, Amjadi N, Moosavi S, Akbari S. Metformin compared with insulin in the management of gestational diabetes mellitus: A randomized clinical trial. *Diabetes Res Clin Pract*. 2012; 98(3): 422-9.
61. Moore LE, Briery CM, Clokey D, Martin RW, Williford NJ, Bofill JA, et al. Metformin and insulin in the management of gestational diabetes mellitus: Preliminary results of a comparison. *J Rep Med Obstet Gynecol*. 2007; 52(11): 1011-5.
62. Mesdaghinia E, Samimi M, Homaei Z, Saberi F, Moosavi SGA, Yaribakht M. Comparison of newborn outcomes in women with gestational diabetes mellitus treated with metformin or insulin: a randomised blinded trial. *Int J Prev Med*. 2013; 4(3): 327-33.
63. Ijäs H, Vääräsmäki M, Morin-Papunen L, Keravuo R, Ebeling T, Saarela T, et al. Metformin should be considered in the treatment of gestational diabetes: a prospective randomised study. BJOG; 2010. p. 880-5.
64. Hague W, Davoren P, Oliver J, Rowan J. Contraindications to use of metformin. Metformin may be useful in gestational diabetes. *BMJ*. 2003; 326: 762.
65. Hassan JA, Karim N, Sheikh Z. Metformin prevents macrosomia and neonatal morbidity in gestational diabetes. *Pak J Med Sci*. 2012; 28(3): 384-9.
66. Ainuddin J, Karim N, Hasan AA, Naqv iSA. Metformin versus insulin treatment in gestational diabetes in pregnancy in a developing country. A randomized control trial. *Diab Res Clin Prac*. 2014.
67. Anjalakshi C, Balaji V, Balaji MS, Seshiah V. A prospective study comparing insulin and glibenclamide in gestational diabetes mellitus in Asian Indian women. *Diabetes Res Clin Pract*. 2007; 76(3): 474-5.
68. Bertini AM, Silva JC, Taborda W, Becker F, Lemos Bebber FR, Zucco Viesi JM, et al. Perinatal outcomes and the use of oral hypoglycemic agents. J perinat med; 2005. p. 519-23.
69. Lain KY, Garabedian MJ, Daftary A, Jeyabalan A. Neonatal adiposity following maternal treatment of gestational diabetes with glyburide compared with insulin. *Am J Obstet Gynecol*. 2009; 200(5): 501.e1-.e6.
70. Langer O, Conway DL, Berkus MD, Xenakis EM-J, Gonzales O. A Comparison of Glyburide and Insulin in Women with Gestational Diabetes Mellitus. *N Engl J Med*. 2000; 343(16): 1134-8.
71. Mukhopadhyay P, Bag TS, Kyal A, Saha DP, Khalid N. Oral hypoglycemic glibenclamide: Can it be a substitute to insulin in the management of gestational diabetes mellitus? a comparative study. *J South Asian Feder Obstet Gynae*. 2012; 4(1): 28-31.
72. Ogunyemi D, Jesse M, Davidson M. Comparison of glyburide versus insulin in management of gestational diabetes mellitus. Endo prac; 2007. p. 427-8.
73. Silva J, Bertini A, Taborda W, F. B, Bebber F, Aquim G, et al. Glibenclamide in the Treatment for Gestational Diabetes Mellitus in a Compared Study to Insulin. *Arq Bras Endocrinol Metab*. 2007; 51(4): 541-6.
74. Tempe A, Mayanglambam RD. Glyburide as treatment option for gestational diabetes mellitus. *J Obstet Gynaecol Res*. 2013; 39(6): 1147-52.
75. Moore LE, Clokey D, Rappaport VJ, Curet LB. Metformin compared with glyburide in gestational diabetes: a randomized controlled trial. Obst Gynecol; 2010. p. 55-9.
76. George A, Mathews JE, Sam D, Beck M, Benjamin SJ, Abraham A, et al. Comparison of neonatal outcomes in women with gestational diabetes with moderate hyperglycaemia on metformin or glibenclamide - A randomised controlled trial. Aust N Z J Obstet Gynaecol; 2015. p. 47-52.
77. Silva JC, Fachin DRlRN, Coral ML, Bertini AM. Perinatal impact of the use of metformin and glyburide for the treatment of gestational diabetes mellitus. *J Perinat Med*. 2012; 40(3): 225-8.
78. Lawlor DA. The Society for Social Medicine John Pemberton Lecture 2011. Developmental overnutrition—an old hypothesis with new importance? *Int J Epidemiol*. 2013; 42 (1): 7-29.
79. O'Sullivan JB, Mahan CM. Criteria for the oral glucose tolerance test in pregnancy. *Diabetes*. 1967; 13: 278–85.

For peer review only - http://bmjopen.bmj.com/site/about/guidelines.xhtml

80. Metzger BE LL, Dyer AR, et al. HAPO Study Cooperative,. Hyperglycemia and Adverse Pregnancy Outcomes. *N Engl J Med*. 2008; 358: 1991-2002.
81. Farrar D, Fairley L, Santorelli G, Tuffnell D, Sheldon TA, Wright J, et al. Association between hyperglycaemia and adverse perinatal outcomes in south Asian and white British women: analysis of data from the Born in Bradford cohort. *Lancet Diabetes Endocrinol*. 2015; 3(10): 795-804.
82. World Health Organization. Diagnostic criteria and classification of hyperglycaemia first detected in pregnancy. *World Health Organisation*. 2013; http://apps.who.int/iris/bitstream/10665/85975/1/WHO_NMH_MND_13.2_eng.pdf?ua=1%2520%2520.

For peer review only

For peer review only - http://bmjopen.bmj.com/site/about/guidelines.xhtml

Table 1: Trials comparing a package of care starting with dietary modification to routine care and trials comparing a dietary modification with another dietary modification

First author	Year	Location	Number	Screening strategy used to determine need for diagnostic test	Diagnostic test and glucose thresholds used to diagnose GDM (mmol/L)	Intervention group	Control group	Insulin use in diet group	In meta-analyses	Meta-analysis outcome
Trials comparing a package of care (starting with dietary modification) to routine care										
Bevier[45]	1999	USA	103	50g OGCT >7.8	Positive OGCT, negative 100g OGTT, levels not reported	Dietary counselling and home monitoring	Routine care	If needed	yes	Apgar 5 min, BW, C-section, GA at birth, induction, instrumental birth, macrosomia, pre-eclampsia, shoulder dystocia
Bonomo[46]	2005	Italy	300	Risk factors and 50g OGCT	Positive OGCT ≥7.8, negative 100g OGTT 'C&C criteria'	Dietary advice and monitoring	Routine care	Not reported	yes	Apgar 5 min, BW, C-section, GA at birth, LGA, macrosomia, NN hypoglycaemia, NICU admission,

For peer review only - http://bmjopen.bmj.com/site/about/guidelines.xhtml

Crowther[47]	2005	UK / Australia	1000	Risk factors or 50g OGCT	75g OGTT fasting <7.8 and 2-hr >7.8 and < 11.1	Individualised dietary advice, monitoring & pharmacological treatments	Routine care	If needed	yes	Apgar 5 min <7, BW, C-sectionGA at birth, induction, macrosomia, NN hypoglycaemia, NICU admission, pre-eclampsia, shoulder dystocia
Deveer[33]	2013	Turkey	100	Universal 50g OGCT >7.8 and <10.0	Positive OGCT, negative 100g OGTT fasting <5.3 1-hr <10.0, 2-hr <8.8 and 3-hr <7.8	Calorie diet	Routine care	Not reported	yes	BW, C-section, gest age at birth, LGA, macrosomia, NICU admission, pre-eclampsia, preterm birth
Elnour[48]	2006	UAE	180	Not reported	100g OGTT, 'C&C criteria'	Diet education, exercise, monitoring & pharmacological treatments	Routine care	If needed	yes	C-section, LGA, macrosomia, NN hypoglycaemia, NICU admission, pre-eclampsia, preterm birth, shoulder dystocia

For peer review only

For peer review only - http://bmjopen.bmj.com/site/about/guidelines.xhtml

Fadl[34]	2015	Sweden	66	Risk factors	75g OGTT <7.0, >10.0 <12.2	Diet education, exercise, monitoring & pharmacological treatments	Routine care	If needed in intervention group only	yes	BW, C-section, LGA, GA at birth, macrosomia, pre-eclampsia, instrumental birth, induction, NICU admission
Garner[49]	1997	Canada	299	75g OGCT >8.0	75g OGTT fasting >7.5 and 2-hr >9.6	Dietary counselling, restricted calorie intake, monitoring & insulin if required	Routine care	If needed	yes	BW, C-section, GA at birth, macrosomia, NN hypoglycaemia, pre-eclampsia, preterm birth, shoulder dystocia
Landon[50]	2009	USA	958	50g OGCT >7.5-<11.1	100g OGTT fasting <5.3, 2 or more 1-hr >8.6 or 2-hr >8.6	Individualised dietary advice, monitoring & insulin	Routine care	If needed	yes	BW, C-section, GA at birth, induction, macrosomia, NN hypoglycaemia, NICU admission, pre-eclampsia, preterm birth, shoulder dystocia

For peer review only

Li[51]	1987	Hong Kong	58	Risk factors	100g OGTT, two or more: fasting >5.8, 1 hr >10.6, 2-hr >9.2, 3-hr >8.1, then 75g OGTT fasting <8.0 or 2-hr <11.0	30-35g/kg carbohydrate diet and monitoring	Routine care	Not reported	yes	BW, C-section, GA at birth, induction, macrosomia,
O'Sullivan[52]	1966	USA	615	OGCT or risk factors	100g OGTT two or more fasting > 6.1, or 1-hr > 9.1 or 2-hr > 6.7 or 3-hr >6.1	Low calorie diabetic diet	Standard diabetic diet	Only in intervention group	yes	Macrosomia, preterm birth
Yang[35]	2003	China	150	Not reported	Not reported	'intensive' diabetes management	Routine care	If needed	yes	C-Section, shoulder dystocia
Yang[36]	2014	China	700		75g OGTT fasting 5.1, 1 hr 10.0, 2 hr 8.5	Individual & group dietary/physical intervention	Routine care	If needed	yes	BW, C-section, GA at birth, induction, macrosomia, NN hypoglycaemia, PIH, pre-eclampsia, preterm birth, shoulder dystocia

Trials comparing a dietary modification with another dietary modification

For peer review only - http://bmjopen.bmj.com/site/about/guidelines.xhtml

Asemi[53]	2014	Iran	52	50g OGCT	OGCT ≥7.8, 75g OGTT Fasting: >5.1, 1 hr ≥10.0, 2 hr ≥8.5	DASH diet[a]	Control diet	Women with GDM excluded, therefore insulin not required	no	-
Cypryk[54]	2007	Poland	30	Not reported	levels not reported only that the WHO criteria was used	High carbohydrate diet	Low carbohydrate diet	If needed	no	-
Louie[55]	2011	Australia	99	Not reported	75g OGTT≥5.5, 1-hr ≥10.0 or 2-h ≥8.0	Low GI diet	High fibre moderate GI diet	If needed	no	-
Ma[37]	2015	China	83	50g OGCT	75g OGTT≥5.8, 1-hr ≥10.6, 2-h ≥9.2 or 3-hr 8.1	Low glycaemia load diet	Usual diet	If needed[b]	no	-
Moreno-Castilla[56]	2013	Spain	152	50g OGCT ≥7.8	100g OGTT >5.8, 1 hr >10.6, 2-hr >9.2, 3-hr >8.1	Low carbohydrate diet	Control diet	If needed	no	-
Rae[57]	2000	Australia	124	Not reported	(glucose load not reported) OGTT fasting >5.4 or 2-hour > 7.9	Calorie restricted diet	Usual diet	If needed	no	-
Yao[38]	2015	China	33	50g OGCT fasting ≥5.8 'post-load' ≥7.8	100g OGTT fasting >5.3, 1 hr >10.0, 2-hr >8.6, 3-hr >7.8	DASH diet	Usual diet	If needed	no	-

[a]DASH diet = Dietary Approaches to Stop Hypertension
[b]women who required insulin were excluded from the trial's analyses

For peer review only

Table 2: Trials comparing metformin to insulin

First author	Year	Location	Number	Diagnostic test and glucose thresholds used to diagnose GDM	Screening strategy[a]	Meta-analysis outcome
Ainuddin[66]	2014	Pakistan	150	75g OGTT two or more; fasting 5.3, 1 hr 10.0, 2 hr 8.6	50g OGCT≥7.8	PIH, pre-eclampsia, GA at delivery, induction, C-section, LGA, NICU admission, neonatal hypoglycaemia
Hague[64]	2003	Australia	30	75g OGTT fasting >5.5 or 2-hr >8.0	Risk factors	BW, Pre-eclampsia, GA at birth, induction, C-section, macrosomia, hypoglycaemia
Hassan[65]	2012	Pakistan	150	75g OGTT 2 or more levels fasting >5.3, 1-hr >10.0 or 2-hr >8.6	50g OGCT ≥7.8	Apgar 5 min, GA at birth, induction, C-section, BW, macrosomia, hypoglycaemia, NICU admission
Ijas[63]	2010	Finland	100	75g OGTT fasting >5.3, 1-hr >11.0 or 2-hr >9.6	Risk-based	Apgar 5 min, BW, C-section, GA at birth, induction, instrumental birth, LGA,

For peer review only - http://bmjopen.bmj.com/site/about/guidelines.xhtml

						macrosomia, hypoglycaemia, NICU admission
Mesdaghinia[62]	2013	Iran	200	100g OGTT two or more; fasting >5.3 or 1-hr >10.0 or 2-hr >8.6 or 3-hr >7.8	50g OGCT–levels not reported	BW, macrosomia, LGA, hypoglycaemia, NICU admission, shoulder dystocia, 5 min Apgar <7, preterm birth
Moore[61]	2007	USA	63	100g OGTT two or more; fasting >5.8 or 1-hr >10.5 or 2-hr >9.1 or 3-hr >8.0	50g OGCT $\geq$7.8	Apgar 5 min, BW, macrosomia, hypoglycaemia, NICU admission
Niromanesh[60]	2012	Iran	160	100g OGTT two or more fasting >5.3, 1-hr >10.0, 2-hr, 3-hr >8.6 or 3-hr >7.8	50g OGCT $\geq$7.2	Apgar 5 min, pre-eclampsia, PIH GA at birth, induction, C-section, shoulder dystocia, BW macrosomia, LGA, NICU admission, hypoglycaemia, preterm birth
Rowan[59]	2008	Australia / NZ	751	75g OGTT fasting >5.5 or 2-hr >8.0	Risk factors	Apgar 5 min <7, BW, GA at birth, LGA, NICU admission, PIH, pre-eclampsia, preterm birth
Spaulonci[58]	2013	Brazil	94	75g or 100g OGTT fasting >5.3 or 1-hr >10.0 or 2-hr >8.0	No screening	GA at birth, BW, Apgar 5 min, macrosomia,

For peer review only

				and two or more fasting >5.3, 1-hr >10.0, 2-hr, 3-hr >8.6 or 3-hr >7.8 respectively		hypoglycaemia, pre-eclampsia, preterm birth, C-section
Tertti[43]	2013	Finland	217	75g OGTT both criteria: fasting ≥4.8, 1-h ≥10.0, 2-h ≥8.7 and fasting ≥5.3, ≥10.0 and ≥8.6 respectively	Risk factors	GA at birth, BW, Apgar at 5 min, induction, instrumental birth, C-section, LGA, macrosomia, preterm birth, PIH, pre-eclampsia, NICU admission, hypoglycaemia
Zinnat[31]	2013	Bangladesh	450	Not reported[d]	Not reported[d]	Macrosomia, shoulder dystocia, C-section, instrumental birth hypoglycaemia, NICU admission,

[a]It is assumed unless otherwise reported, that the screening strategy advocated by the criteria used was adhered to
[d]Conference abstract

For peer review only

For peer review only - http://bmjopen.bmj.com/site/about/guidelines.xhtml

Table 3: Trials comparing glibenclamide (glyburide) to insulin

First author	Year	Location	Number	Diagnostic test and glucose thresholds used to diagnose GDM	Screening strategy[a]	Outcome
Anjalakshi[67]	2007	India	23	75g OGTT 2-hr >7.8	Universal OGTT	BW
Bertini[68]	2005	Brazil	70	75g OGTT fasting >6.1 or 2-hr >7.8	Not reported	BW, C-section, Apgar 5 min, GA at birth, LGA
Lain[69]	2009	USA	99	100g OGTT 2 or more: fasting >5.3, 1-hr >8.6 or 2-hr >8.6	50g >7.5	BW, GA at birth, LGA, macrosomia
Langer[70]	2000	USA	404	100g OGTT fasting >5.3-<7.8	50g OGCT >7.3	BW, C-section, GA at birth, LGA, macrosomia, hypoglycaemia, NICU admission, pre-eclampsia
Mirzamoradi[32]	2015	Iran	96	Glucose load not reported; OGTT 2 or more: fasting >5.3, 1-hr >10.0, 2-hr >8.3	Universal OGTT	BW, C-section, GA at birth, NICU admission, hypoglycaemia, pre-eclampsia
Mukhopadhyay[71]	2012	India	60	75g OGTT 2-hr >7.8	No screening	BW, GA at birth, LGA, hypoglycaemia
Ogunyemi[72]	2007	USA	97	Not reported	Not reported	BW, C-section, GA at birth, hypoglycaemia,

For peer review only

For peer review only - http://bmjopen.bmj.com/site/about/guidelines.xhtml

Silva[73]	2007	Brazil	68	75g OGTT fasting >6.1 or 2-hr >7.8	No screening	BW, C-section, LGA, macrosomia,
Tempe[74]	2013	India	64	100g OGTT 2 or more: fasting >5.3, 1-hr >10.0, 2-hr >8.6 or 3-hr >7.8	50g OGCT >7.2	BW, GA birth, macrosomia, hypoglycaemia, NICU admission, pre-eclampsia, preterm birth

[a]It is assumed unless otherwise reported, that the screening strategy advocated by the criteria used was adhered to

For peer review only - http://bmjopen.bmj.com/site/about/guidelines.xhtml

Table 4: Trials comparing glibenclamide to metformin

First author	Year	Location	Number	Diagnostic test and thresholds used to diagnose GDM (mmol/L)	Screening strategy[a]	Outcome
George[76]	2015	India	159	100g OGTT 2 or more; fasting ≥5.3 or 1 hr ≥10.0 or 2-hr ≥8.6	Not reported	BW, GA at birth, macrosomia, hypoglycaemia
Moore[75]	2010	USA	149	100g OGTT 2 or more; fasting >5.3 or 2-hr >6.7	50g OGCT>7.2	BW, C-section, GA at birth, macrosomia, hypoglycaemia, NICU admission, pre-eclampsia, shoulder dystocia
Silva[77]	2012	Brazil	200	75g OGTT fasting >5.3 or 1-hr >10.0 or 2-hr >8.0	No screening	Apgar 5 min, BW, C-section, GA at birth, LGA, macrosomia, hypoglycaemia, NICU admission

For peer review only - http://bmjopen.bmj.com/site/about/guidelines.xhtml

Table 5: Estimated probability (%) of a treatment being the most effective in reducing the risk of a dichotomous outcome.

	Treatment		
	Insulin	Metformin	Glibenclamide (Glyburide)
**Outcome**			
LGA	7.1	92.8	0.1
Macrosomia	5.6	94.0	0.3
Neonatal intensive care admission	0.5	61.2	38.3
Neonatal hypoglycaemia	3.3	96.3	0.4
Caesarean section	10.4	9.7	79.9
Pre-eclampsia	4.8	84.0	11.2

Figure 1: Search process

Figure 2: Forest plots for treatment comparisons and perinatal outcomes
2a: Packages of care (starting with dietary modification) versus routine care: dichotomous outcomes
2b: Packages of care (starting with dietary modification) versus routine care: continuous outcomes
2c: Metformin versus insulin: dichotomous outcomes
2d: Metformin versus insulin: continuous outcomes
2e: Glibenclamide versus insulin: dichotomous outcomes
2f: Glibenclamide versus insulin: continuous outcomes
2g: Glibenclamide versus metformin: dichotomous outcomes
2h: Glibenclamide versus metformin: continuous outcomes

Figure 3: Network meta-analysis, relationship of treatment comparisons

Figure 4: Network meta-analysis comparing metformin, glibenclamide and insulin. First better – treatment listed first in the outcome column is superior; second better – treatment listed second in the outcome column is superior.

Supplementary Figure 1: Dietary modification trials: dichotomous outcomes

Supplementary Figure 2: Dietary modification trials: continuous outcomes

For peer review only - http://bmjopen.bmj.com/site/about/guidelines.xhtml

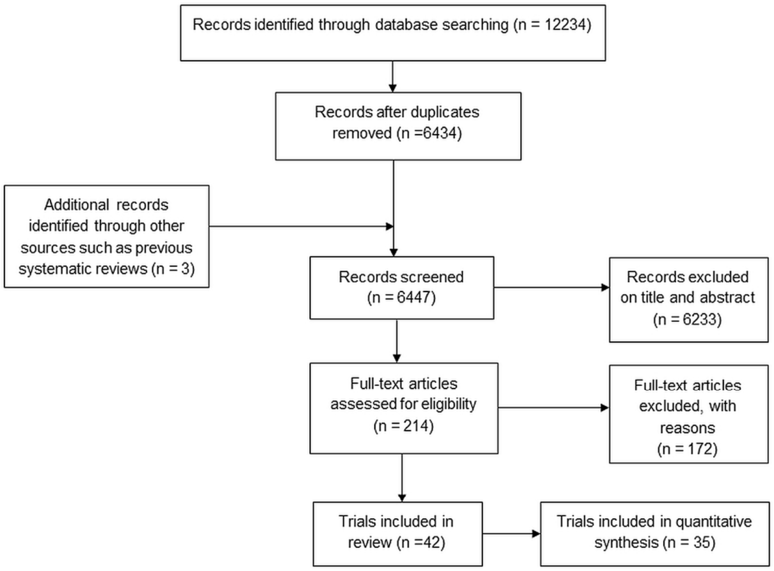

Figure 1: Search process

127x95mm (300 x 300 DPI)

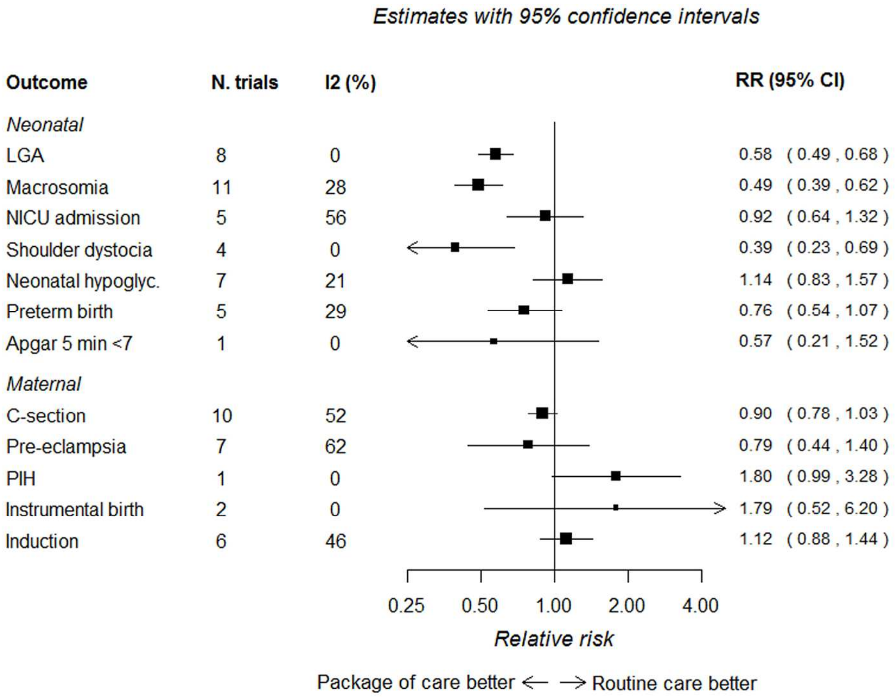

Figure 2a: Packages of care (starting with dietary modification) versus routine care: dichotomous outcomes

127x103mm (300 x 300 DPI)

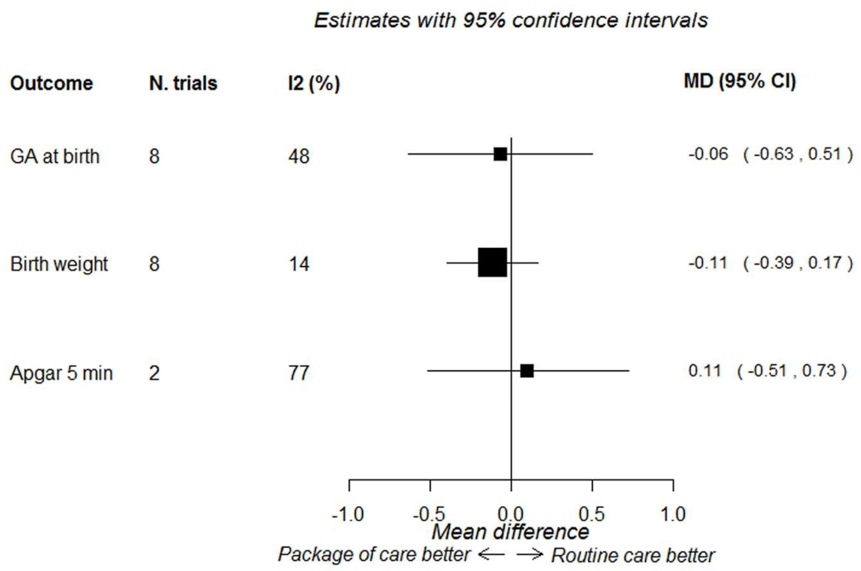

Figure 2b: Packages of care (starting with dietary modification) versus routine care: continuous outcomes

127x85mm (300 x 300 DPI)

view only

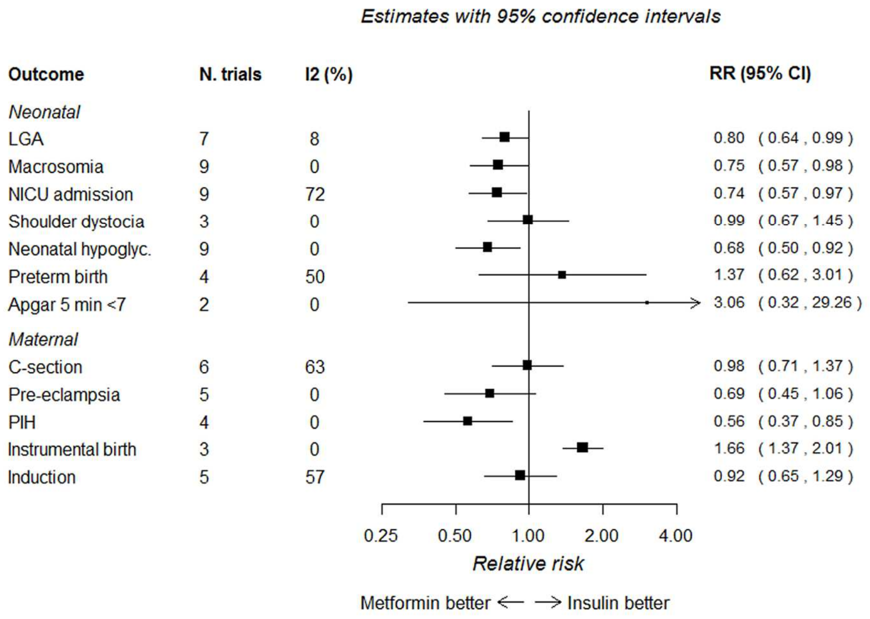

Figure 2c: Metformin versus insulin: dichotomous outcomes

127x91mm (300 x 300 DPI)

For peer review only - http://bmjopen.bmj.com/site/about/guidelines.xhtml

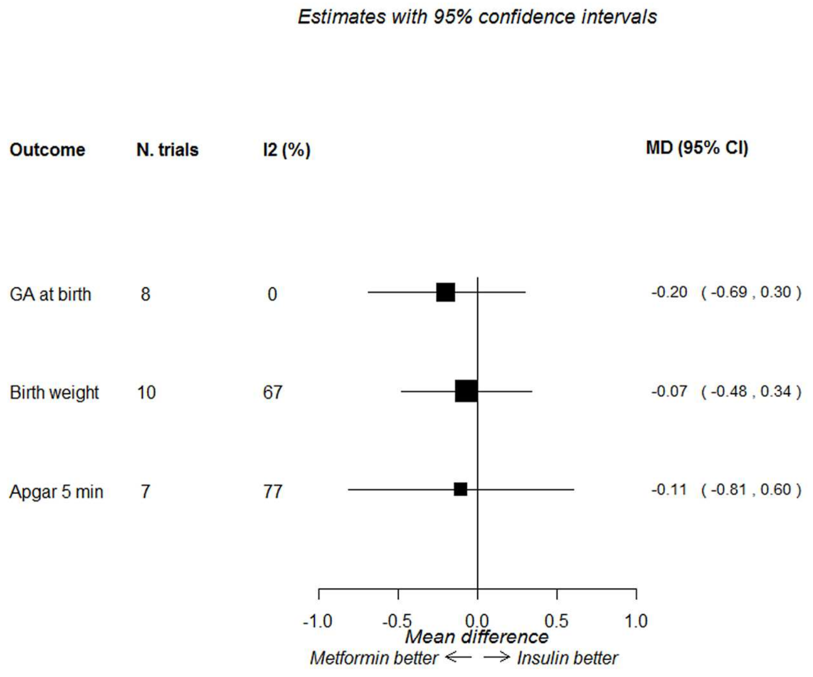

Figure 2d: Metformin versus insulin: continuous outcomes

127x107mm (300 x 300 DPI)

For peer review only

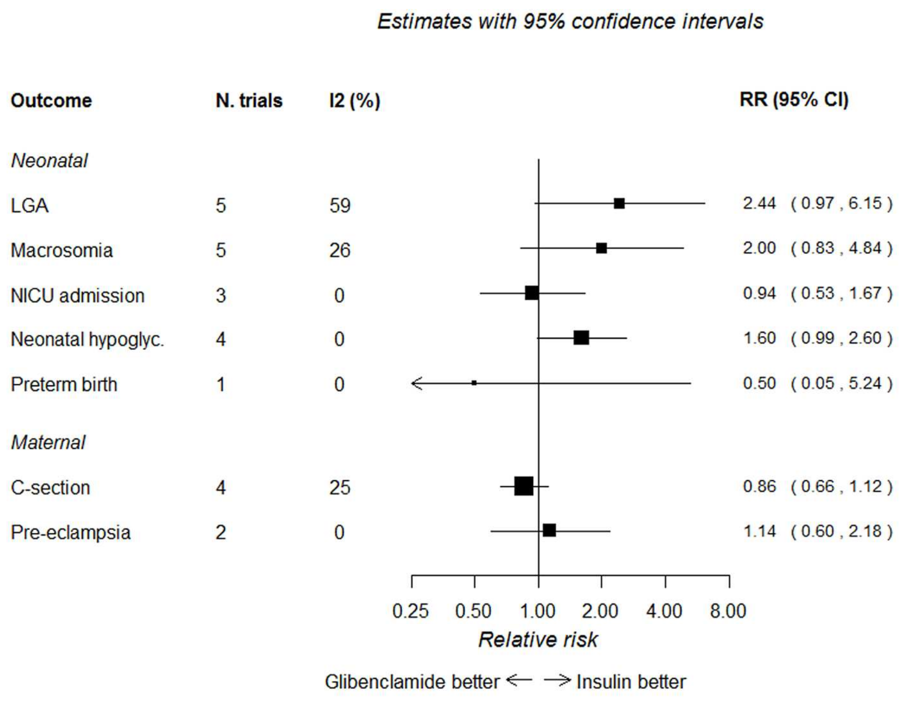

Figure 2e: Glibenclamide versus insulin: dichotomous outcomes

127x103mm (300 x 300 DPI)

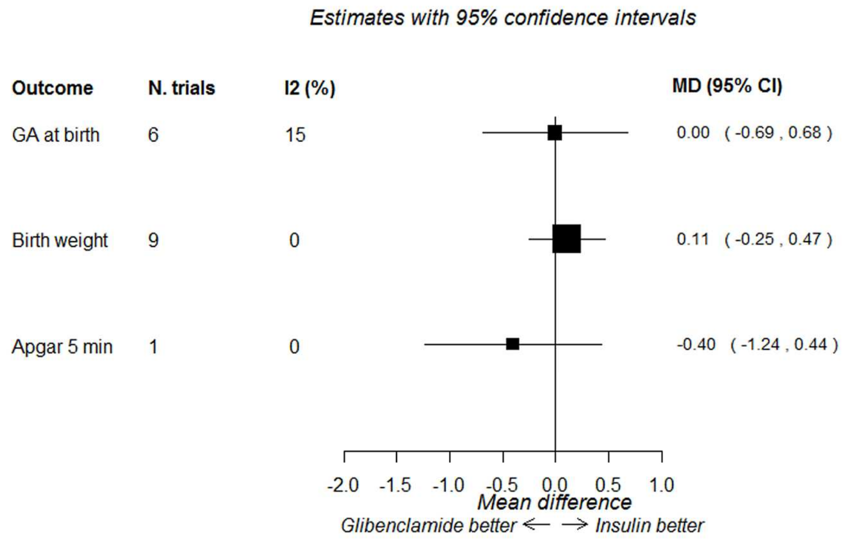

Figure 2f: Glibenclamide versus insulin: continuous outcomes

127x79mm (300 x 300 DPI)

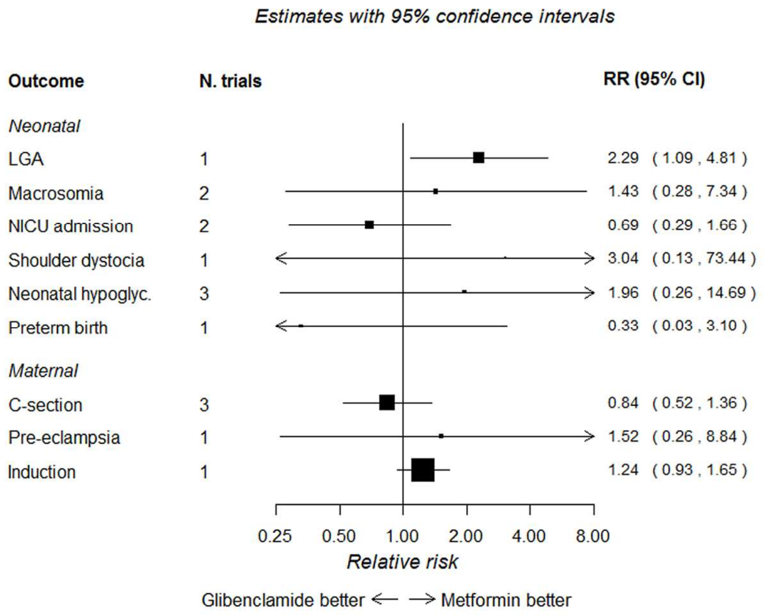

Figure 2g: Glibenclamide versus metformin: dichotomous outcomes

127x91mm (300 x 300 DPI)

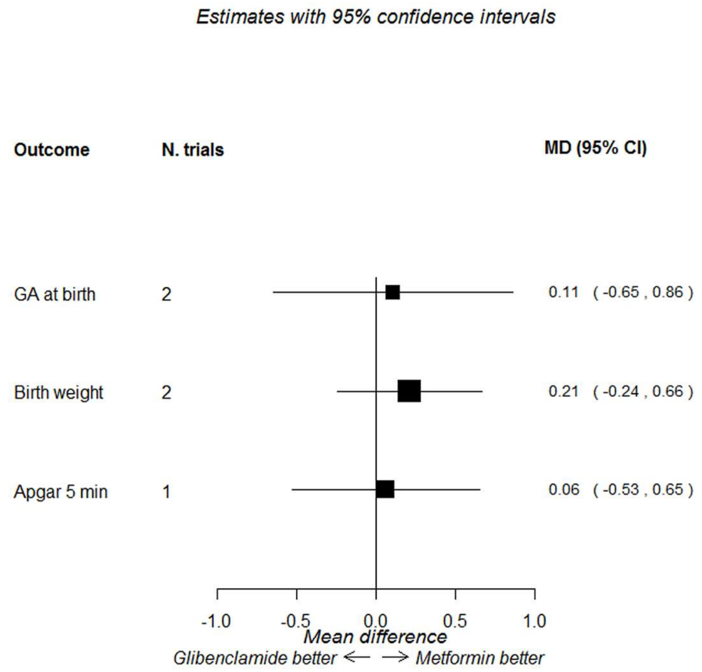

Figure 2h: Glibenclamide versus metformin: continuous outcomes

127x107mm (300 x 300 DPI)

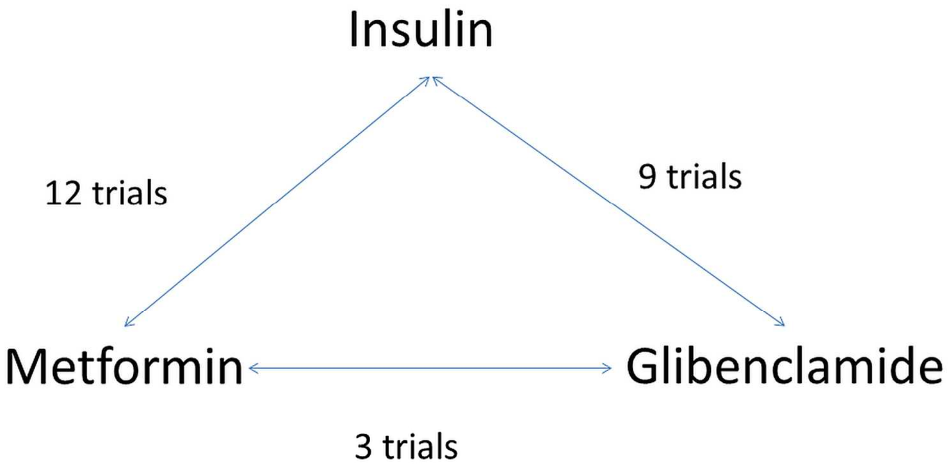

Figure 3: Network meta-analysis, relationship of treatment comparisons

127x95mm (300 x 300 DPI)

For peer review only - http://bmjopen.bmj.com/site/about/guidelines.xhtml

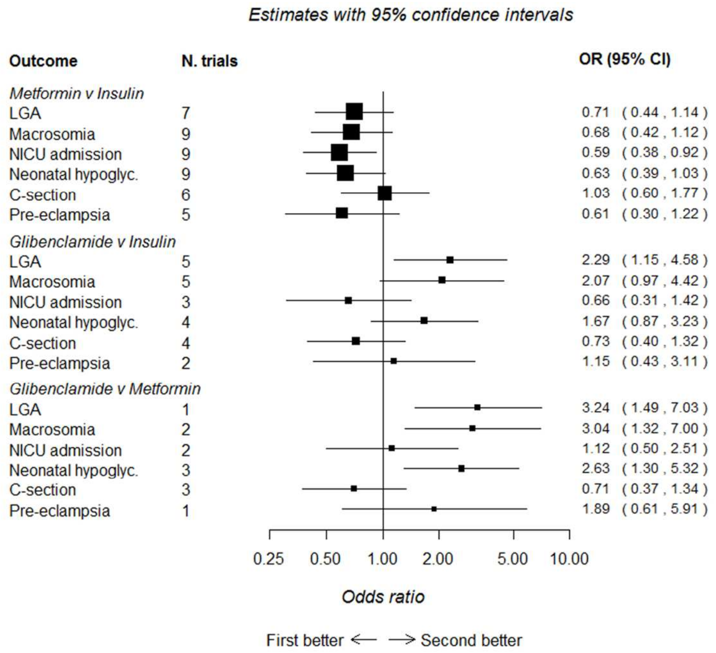

Figure 4: Network meta-analysis comparing metformin, glibenclamide and insulin. First better – treatment listed first in the outcome column is superior; second better – treatment listed second in the outcome column is superior.

127x96mm (300 x 300 DPI)

w only

Supplementary file 1: Search strategy

1 exp diabetes, gestational/ (8715)
2 (gestation$ adj4 diabet$).ti,ab. (10162)
3 gdm.ti,ab. (4203)
4 (glucose adj4 (pregnan$ or gestation$ or prenatal$ or antenatal$ or pre-natal$ or ante-natal$ or maternal$)).ti,ab. (3796)
5 or/1-4 (15126)
6 Glucose Intolerance/ (7142)
7 Glucose Tolerance Test/ (31300)
8 IGT.ti,ab. (4074)
9 ((impair$ or reduced) adj2 glucose).ti,ab. (19442)
10 (glucose adj (tolerance$ or intolerance$)).ti,ab. (40791)
11 (gtt or ogtt).ti,ab. (7907)
12 Prediabetic State/ (4763)
13 (prediabet$ or pre-diabet$).ti,ab. (6103)
14 exp Insulin Resistance/ (64450)
15 (metabolic syndrome$ or syndrome$ x or borderline diabet$).ti,ab. (37636)
16 or/6-15 (134039)
17 exp Pregnancy/ (795751)
18 (pregnan$ or gestation$ or prenatal$ or antenatal$ or pre-natal$ or ante-natal$ or maternal$).ti,ab. (639369)
19 or/17-18 (1008161)
20 16 and 19 (10229)
21 5 or 20 (20405)
22 randomized controlled trial.pt. (421926)
23 controlled clinical trial.pt. (91079)
24 random$.ti,ab. (841233)
25 placebo.ti,ab. (176519)
26 drug therapy.fs. (1876752)
27 trial.ti,ab. (430134)
28 groups.ab. (1574965)
29 or/22-28 (3970247)
30 21 and 29 (6337)
31 (2014$ or 2015$ or 2016$).ed,dc,dp,ep,vd,yr. (3346601)
32 30 and 31 (1671)
33 animals/ not humans/ (4235813)
34 32 not 33 (1555)

For peer review only

Supplementary Table 1: Quality assessment of the included trials

Author	Year	Random sequence generation	Allocation concealment	Blinding of participants	Blinding of outcome assessments	Completeness of outcome data	Selective reporting
Ainuddin[1]	2015	unclear	high risk	high risk	unclear	low risk	unclear
Anjalakshi[2]	2007	unclear	unclear	unclear	unclear	low risk	unclear
Asemi[3]	2014	low risk	unclear	high risk	high risk	low risk	low risk
Bertini[5]	2005	low risk	low risk	high risk	high risk	low risk	low risk
Bevier[6]	1999	unclear	unclear	high risk	high risk	high risk	low risk
Bonomo[7]	2005	unclear	unclear	high risk	high risk	low risk	unclear
Crowther[8]	2005	low risk	low risk	high risk	low risk	low risk	low risk
Cypryk[9]	2007	unclear	high risk	unclear	unclear	low risk	high risk
Deveer[10]	2013	high risk	high risk	high risk	high risk	low risk	low risk
Elnour[11]	2008	unclear	high risk	high risk	high risk	high risk	low risk
Fadl[12]	2015	Low risk	low risk	unclear	unclear	low risk	unclear
Garner[13]	1997	low risk	high risk	high risk	high risk	low risk	low risk
George[14]	2015	low risk	high risk	high risk	unclear	low risk	low risk

For peer review only

Author	Year	Random sequence generation	Allocation concealment	Blinding of participants	Blinding of outcome assessments	Completeness of outcome data	Selective reporting
Hague[15]	2003	unclear	unclear	unclear	unclear	unclear	unclear
Hassan[16]	2012	high risk	high risk	unclear	unclear	low risk	low risk
Ijas[17]	2010	low risk	low risk	high risk	high risk	low risk	low risk
Lain[18]	2009	low risk	low risk	low risk	low risk	high risk	low risk
Landon[19]	2009	low risk	low risk	high risk	low risk	low risk	low risk
Langer[20]	2000	low risk	unclear	unclear	unclear	low risk	low risk
Li[21]	1987	high risk	unclear	high risk	unclear	low risk	low risk
Louie[22]	2011	low risk	low risk	low risk	unclear	low risk	high risk
Ma[23]	2015	high risk	high risk	high risk	unclear	low risk	unclear
Mesdaghinia[24]	2012	low risk	low risk	low risk	low risk	low risk	low risk
Mirzamoradi[25]	2015	unclear	unclear	high risk	unclear	low risk	unclear
Moore[26]	2007	low risk	unclear	unclear	unclear	low risk	low risk
Moore[27]	2010	low risk	low risk	high risk	high risk	low risk	low risk
Moreno-Castilla[28]	2013	unclear	low risk	high risk	unclear	low risk	low risk
Mukhopadhyay[29]	2012	low risk	unclear	unclear	unclear	low risk	low risk

For peer review only

Author	Year	Random sequence generation	Allocation concealment	Blinding of participants	Blinding of outcome assessments	Completeness of outcome data	Selective reporting
Niromanesh[30]	2012	low risk	low risk	unclear	low risk	low risk	low risk
Ogunyemi[31]	2007	low risk	unclear	unclear	unclear	low risk	unclear
O'Sullivan[32]	1966	unclear	unclear	high risk	high risk	unclear	unclear
Rae[33]	2000	unclear	unclear	low risk	unclear	low risk	high risk
Rowan[34]	2008	low risk	unclear	high risk	high risk	low risk	low risk
Silva[35]	2012	low risk	unclear	high risk	high risk	low risk	low risk
Silva[36]	2007	unclear	low risk	high risk	high risk	low risk	low risk
Spaulonci[37]	2013	low risk	unclear	unclear	unclear	low risk	low risk
Tempe[38]	2013	unclear	unclear	unclear	unclear	low risk	low risk
Tertti[39]	2013	unclear	unclear	unclear	unclear	low risk	low risk
Yang[40]	2014	unclear	high risk	low risk	high risk	low risk	unclear
Yang[41]	2003	unclear	unclear	high risk	unclear	high risk	unclear
Yao[42]	2015	unclear	unclear	unclear	unclear	Low risk	unclear
Zinnat[43]	2013	unclear	unclear	unclear	unclear	low risk	unclear

For peer review only

▲ = Alwan review- publications identified by their 2011 search and awaiting classification

For peer review only

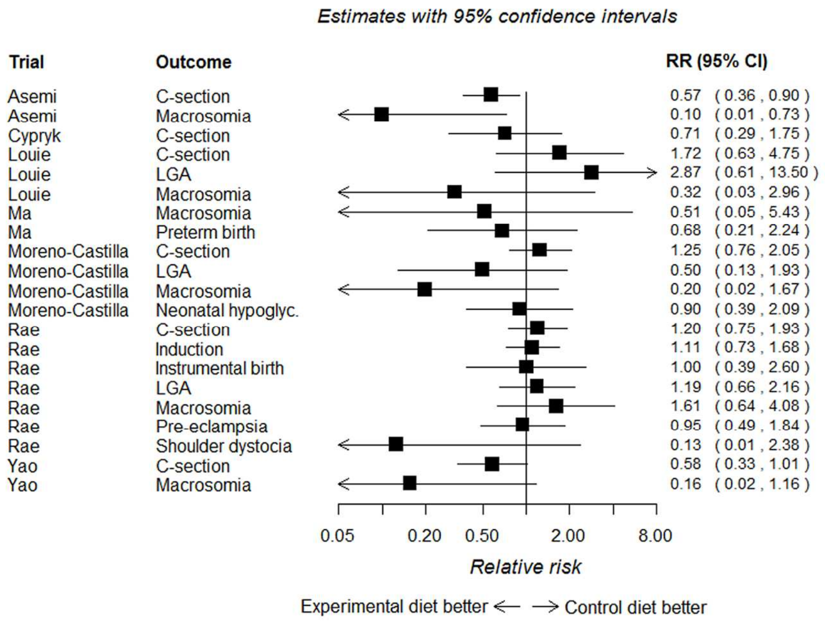

Supplementary Figure 1: Dietary modification trials: dichotomous outcomes

127x91mm (300 x 300 DPI)

For peer review only

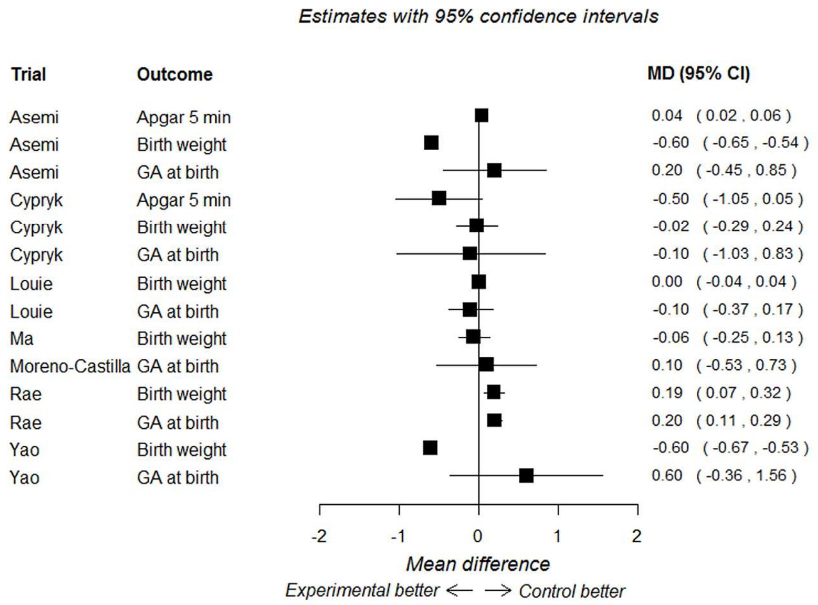

Supplementary Figure 2: Dietary modification trials: continuous outcomes

127x91mm (300 x 300 DPI)

For peer review only

PRISMA 2009 Checklist

Section/topic	#	Checklist item	Reported on page #
**TITLE**			
Title	1	Identify the report as a systematic review, meta-analysis, or both.	1
**ABSTRACT**			
Structured summary	2	Provide a structured summary including, as applicable: background; objectives; data sources; study eligibility criteria, participants, and interventions; study appraisal and synthesis methods; results; limitations; conclusions and implications of key findings; systematic review registration number.	2-3
**INTRODUCTION**			
Rationale	3	Describe the rationale for the review in the context of what is already known.	4
Objectives	4	Provide an explicit statement of questions being addressed with reference to participants, interventions, comparisons, outcomes, and study design (PICOS).	4-5
**METHODS**			
Protocol and registration	5	Indicate if a review protocol exists, if and where it can be accessed (e.g., Web address), and, if available, provide registration information including registration number.	2
Eligibility criteria	6	Specify study characteristics (e.g., PICOS, length of follow-up) and report characteristics (e.g., years considered, language, publication status) used as criteria for eligibility, giving rationale.	6
Information sources	7	Describe all information sources (e.g., databases with dates of coverage, contact with study authors to identify additional studies) in the search and date last searched.	5
Search	8	Present full electronic search strategy for at least one database, including any limits used, such that it could be repeated.	5 & supp file 1
Study selection	9	State the process for selecting studies (i.e., screening, eligibility, included in systematic review, and, if applicable, included in the meta-analysis).	6
Data collection process	10	Describe method of data extraction from reports (e.g., piloted forms, independently, in duplicate) and any processes for obtaining and confirming data from investigators.	6
Data items	11	List and define all variables for which data were sought (e.g., PICOS, funding sources) and any assumptions and simplifications made.	6
Risk of bias in individual studies	12	Describe methods used for assessing risk of bias of individual studies (including specification of whether this was done at the study or outcome level), and how this information is to be used in any data synthesis.	6 & supp table 1
Summary measures	13	State the principal summary measures (e.g., risk ratio, difference in means).	7 - 8
Synthesis of results	14	Describe the methods of handling data and combining results of studies, if done, including measures of consistency (e.g., I^2) for each meta-analysis.	7 - 8

For peer review only - http://bmjopen.bmj.com/site/about/guidelines.xhtml

PRISMA 2009 Checklist

Section/topic	#	Checklist item	Reported on page #
Risk of bias across studies	15	Specify any assessment of risk of bias that may affect the cumulative evidence (e.g., publication bias, selective reporting within studies).	Sup Table 1
Additional analyses	16	Describe methods of additional analyses (e.g., sensitivity or subgroup analyses, meta-regression), if done, indicating which were pre-specified.	-
**RESULTS**			
Study selection	17	Give numbers of studies screened, assessed for eligibility, and included in the review, with reasons for exclusions at each stage, ideally with a flow diagram.	8, figure 1and Table 1
Study characteristics	18	For each study, present characteristics for which data were extracted (e.g., study size, PICOS, follow-up period) and provide the citations.	8 - 9
Risk of bias within studies	19	Present data on risk of bias of each study and, if available, any outcome level assessment (see item 12).	9 supp Table 1
Results of individual studies	20	For all outcomes considered (benefits or harms), present, for each study: (a) simple summary data for each intervention group (b) effect estimates and confidence intervals, ideally with a forest plot.	-
Synthesis of results	21	Present results of each meta-analysis done, including confidence intervals and measures of consistency.	8-13 Figures 2-9
Risk of bias across studies	22	Present results of any assessment of risk of bias across studies (see Item 15).	Supp Table 1
Additional analysis	23	Give results of additional analyses, if done (e.g., sensitivity or subgroup analyses, meta-regression [see Item 16]).	Table 10
**DISCUSSION**			
Summary of evidence	24	Summarize the main findings including the strength of evidence for each main outcome; consider their relevance to key groups (e.g., healthcare providers, users, and policy makers).	13-16
Limitations	25	Discuss limitations at study and outcome level (e.g., risk of bias), and at review-level (e.g., incomplete retrieval of identified research, reporting bias).	15-16
Conclusions	26	Provide a general interpretation of the results in the context of other evidence, and implications for future research.	13-15
**FUNDING**			
Funding	27	Describe sources of funding for the systematic review and other support (e.g., supply of data); role of funders for the systematic review.	17

For peer review only - http://bmjopen.bmj.com/site/about/guidelines.xhtml

PRISMA 2009 Checklist

From: Moher D, Liberati A, Tetzlaff J, Altman DG, The PRISMA Group (2009). Preferred Reporting Items for Systematic Reviews and Meta-Analyses: The PRISMA Statement. PLoS Med 6(6): e1000097. doi:10.1371/journal.pmed1000097

For more information, visit: **www.prisma-statement.org**.

For peer review only

For peer review only - http://bmjopen.bmj.com/site/about/guidelines.xhtml
